# Sensing Volatile Pollutants with Spin-Coated Films Made of Pillar[5]arene Derivatives and Data Validation via Artificial Neural Networks

**DOI:** 10.1021/acsami.4c06970

**Published:** 2024-06-05

**Authors:** Ahmed Nuri Kursunlu, Yaser Acikbas, Ceren Yilmaz, Mustafa Ozmen, Inci Capan, Rifat Capan, Kemal Buyukkabasakal, Ahmet Senocak

**Affiliations:** † Department of Chemistry, Faculty of Science, University of Selcuk, 42250 Konya, Türkiye; ‡ Department of Materials Science and Nanotechnology Engineering, Faculty of Engineering, University of Usak, 64200 Usak, Türkiye; § Department of Physics, Faculty of Science, University of Balikesir, 10145 Balikesir, Türkiye; ∥ Department of Electrical and Electronics Engineering, Faculty of Engineering, University of Usak, 64200 Usak, Türkiye; ⊥ Department of Chemistry, Gebze Technical University, 41400 Gebze, Kocaeli, Türkiye

**Keywords:** pillar[5]arene, surface plasmon resonance, spun thin film, chemical sensor, NARX-ANN model

## Abstract

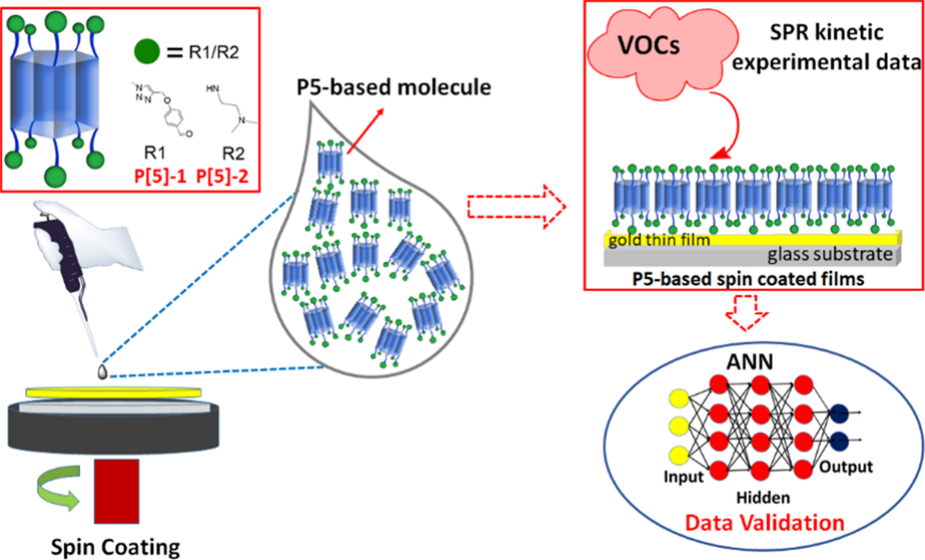

Different types of solvents, aromatic and aliphatic, are used in many industrial sectors, and long-term exposure to these solvents can lead to many occupational diseases. Therefore, it is of great importance to detect volatile organic compounds (VOCs) using economic and ergonomic techniques. In this study, two macromolecules based on pillar[5]arene, named **P[5]-1** and **P[5]-2**, were synthesized and applied to the detection of six different environmentally volatile pollutants in industry and laboratories. The thin films of the synthesized macrocycles were coated by using the spin coating technique on a suitable substrate under optimum conditions. All compounds and the prepared thin film surfaces were characterized by NMR, Fourier transform infrared (FT-IR), elemental analysis, atomic force microscopy (AFM), scanning electron microscopy (SEM), and contact angle measurements. All vapor sensing measurements were performed via the surface plasmon resonance (SPR) optical technique, and the responses of the **P[5]-1** and **P[5]-2** thin-film sensors were calculated with Δ*I*/*I*_o_ × 100. The responses of the **P[5]-1** and **P[5]-2** thin-film sensors to dichloromethane vapor were determined to be 7.17 and 4.11, respectively, while the responses to chloroform vapor were calculated to be 5.24 and 2.8, respectively. As a result, these thin-film sensors showed a higher response to dichloromethane and chloroform vapors than to other harmful vapors. The SPR kinetic data for vapors validated that a nonlinear autoregressive neural network was performed with exogenous input for the best molecular modeling by using normalized reflected light intensity values. It can be clearly seen from the correlation coefficient values that the nonlinear autoregressive with exogenous input artificial neural network (NARX-ANN) model for dichloromethane converged more successfully to the experimental data compared to other gases. The correlation coefficient values of the dichloromethane modeling results were approximately 0.99 and 0.98 for **P[5]-1** and **P[5]-2** thin-film sensors, respectively.

## 1 Introduction

Many industrial products used in our daily life release vapors of volatile organic compounds (VOCs) because of developments in industry and technology. These VOCs threaten human health and create many environmental problems. However, it is not possible to remove them from modern life. To have a healthy life and a clean environment, measuring and balancing the amount of VOCs in the atmosphere is inevitable. Therefore, it has become an important issue to synthesize and fabricate sensor chip materials that can be used to measure the amounts of released VOCs. Many scientists have been trying to synthesize a novel sensor material that is highly sensitive, with short response and recovery times, stable, suitable for multiple uses, selective, and cost-effective because of harmful effects against human beings and environmental pollution. In recent years, new material design and development as a sensor chip have become a complex and challenging field of supramolecular chemistry.^1,2^

A sensor material converts chemical information into a useful analytical signal and has two main considerations: molecular recognition and signal transduction. A sensitive part of the sensor material interacts with the target molecule, whereas the transducer transforms the recognition process into a measurable signal, such as optical, electrical, and magnetic properties, color, and mass change. The fabrication of a thin-film sensor chip that can detect vapor molecules is an important task in science and technology. For molecular recognition, several thin film fabrication methods, such as spin coating,^3^ electrostatic layer-by-layer (LbL) deposition,^4^ and Langmuir–Blodgett (LB) thin film fabrication,^5^ are used. The spin coating technique has been used for several decades for the gas sensor application of thin films due to the control over film thickness, simplicity of the process, low fabrication cost, and no-annealing requirements. The fabrication process generally involves four steps: dispense stage, solid substrate acceleration stage, substrate spinning stage at a constant spin speed, and solvent evaporation stage, which dominates the coating of the material onto the substrate as a thin film formation. Final film thickness of a spin coating film depends on the rotation speed.

On the other hand, chemical information recorded between a sensor chip and a vapor molecule should be converted to an analytical signal using a signal transduction method. To obtain the real-time sensor response during the sensor and vapor interaction, surface plasmon resonance (SPR),^6^ quartz crystal microbalance (QCM),^5^ and fluorescence spectroscopy^7,8^ are used in the field of sensor applications. The SPR technique, which is one of the main optical techniques, is commonly preferred to use due to a higher level of sensitivity, selectivity, and stability when compared with other measurement techniques.^9^ This technique enables us to study the molecular interactions between the thin-film sensing material and the target molecules, which is used to determine the sensor performance in the field of sensor applications. The SPR signal is highly sensitive to refractive index and film thickness changes. This allows a fast online real-time detection with relatively short response times.^10^ In addition, the SPR technique is suitable to develop a low-cost, high-resolution, fast-response chemical sensor. In this study, the SPR method is preferred because it is one of the most sensitive detection methods for changes of thicknesses and refractive indices in ultrathin films. SPR curve measurements are used to calculate the thickness and refractive index of the **P[5]-1** and **P[5]-2** thin-film sensors, and real-time SPR kinetic measurements are used to determine the sensor parameters for the **P[5]-1** and **P[5]-2** thin-film sensors.

It has been mentioned before that the design and synthesis of new synthetic macrocycle materials have resulted in the rapid development of supramolecular chemistry and their applications.^11,12^ Detection of VOCs with chemically based sensors has been realized with macromolecules having an appropriate molecular gap. Among these macromolecules, porphyrins,^13^ phthalocyanines,^14^ cucurbiturils,^15^ crown ethers,^16,17^ cyclodextrins,^18^ and calix[*n*]arenes^19−21^ are the most reported macromolecular-based chemical sensors. A new generation compound in supramolecular science for macrocyclic sensing materials is called pillararenes, which was discovered by Ogoshi.^22^ Pillar[5]arene and pillar[6]arene sensing materials containing five or six repeating units with 10 and 12 substituent groups were synthesized. The position-selective versatile functionalization of these substituents enables the preparation of various supramolecular assemblies.^23,24^ For example, this simple structure of pillararenes containing planar chirality is compatible with outstanding host–guest interactions, which are required for sensor applications. A pillar-shaped architecture has another advantage over pillar[5]arene and pillar[6]arene materials because they have highly symmetrical structures that show regular cyclic pentagonal and hexagonal structures. The construction of various pillararene-based supramolecular assemblies can be easily fabricated because of their outstanding characteristics and versatile structure. As a result, they have become an alternative material in supramolecular science applications.^25,26^ Owing to their easy synthesis, postfunctionalization of the parent pillararene, ring structure, hybridization with inorganic materials, excellent host–guest interaction performance, and physiochemical and thermal stability, they are preferred for use in several applications, such as sensors,^1,27^ biomedical,^28,29^ and environmental applications in the fields of chemistry, physics, biology, and medicine.^30−32^

In the literature, most sensor studies using pillararene materials are concentrated on biomedicine.^33,34^ The number of studies on the study of pillararene materials against VOCs is quite limited.^5−7^ Pillararene sensors were developed by Feng’s group for fluorescence detection of *n*-alkane vapors (hexane, pentane, and heptane) owing to host–guest interactions.^7^ The first investigation of a novel pillar[5]arene-quinoline (P5-Q) molecule as an LB film sensor for the detection of various vapors, such as benzene, toluene, dichloromethane, and chloroform, was reported by Kursunlu et al.^5^ It was found that the host–guest interaction and appropriate cavity of P5-Q caused the encapsulation of these organic compounds. In addition, the dichloromethane vapor response of the P5-Q LB film sensor yielded the highest response because of its lowest molar volume. This has explained that dichloromethane vapors are more mobile and diffuse more easily into the P5-Q LB film than do the others. Another pillar[5]arene-biphenylcarboxylic acid (P5-BPCA) molecule as an LB film sensor was tested against various haloalkane and aromatic hydrocarbon vapors using QCM and SPR methods.^6^ The P5-BPCA LB thin film has better sensitivity and selectivity to dichloromethane vapor than all other haloalkane and aromatic hydrocarbon vapors. The results showed that a higher vapor pressure, dipole moment, and lower molar volume of vapor molecules yield a large SPR response. It is concluded that dipole moment and molar volume play important roles in the vapor sensing interactions between the P5-BPCA LB film and the organic vapor molecules, which is attributed to dipole–dipole interactions or hydrogen bonding.

In recent years, advancements in machine learning tools have spurred an increase in the mathematical modeling of experimental data across various fields. Analytical expressions derived from mathematical models play a crucial role in understanding and analyzing complex processes. Among the array of modeling tools available, artificial neural networks (ANNs) have emerged as a prominent choice. ANNs possess the unique capability to model both linear and nonlinear data, making them highly versatile and adaptable to diverse research contexts. This inherent flexibility has led to widespread preference for ANNs among researchers seeking to model intricate relationships and phenomena within experimental data sets. Consequently, ANNs have become indispensable tools in the arsenal of mathematical modelers, enabling deeper insights and more accurate predictions across a wide range of disciplines.

From predicting material properties and optimizing manufacturing processes to simulating fluid dynamics and analyzing sensor data, ANNs offer a versatile framework for capturing the intricate interplay of variables and phenomena encountered in experimental studies. For instance, Landolsi et al.^35^ designed an ANN for obtaining the response of a metal ion sensor, while Kouda et al.^36^ utilized ANNs for modeling the behavior of an industrial gas sensor. Gugliandolo et al.^37^ presented a model for microwave sensors used in dielectric liquid characterization, and Aswani et al.^38^ employed ANNs for modeling thin-film capacitive humidity sensors.

Based on our previous studies, the idea that increasing the number of donor atoms on the macroring will make the detection of volatile pollutants more effective has gained meaning. Thus, while the cage becomes more open to interaction with increasing donor atoms, it has a wider trap with the carbon chain attached to the main skeleton. For this aim, we designed two electronic cages made with triazole fragments or a diamine derivative. Both the macrocycles include multidonor atoms, such as oxygen and nitrogen, and this situation produced more advantageous results for thin film applications in the detection of VOCs, as explained in the experimental data. A thin-film sensor chip is produced by using the spin coating thin-film fabrication process, and the VOC sensor properties of these new pillar[5]arene materials are investigated by using SPR measurements. The main purpose of this review is to detail the synthesis steps of novel pillar[5]arenes, the thin film production stage, and their reactions to VOC vapors. Furthermore, the accuracy of the performance of P[5]-based sensors was tested using the nonlinear autoregressive with exogenous input artificial neural network (NARX-ANN) model. Trained and experimental data were compared during the validation process. The results showed that the trained data successfully converged with the experimental data, especially for dichloromethane.

## 2 Experimental Details

### 2.1 Materials and Instruments

**Scheme 1:**
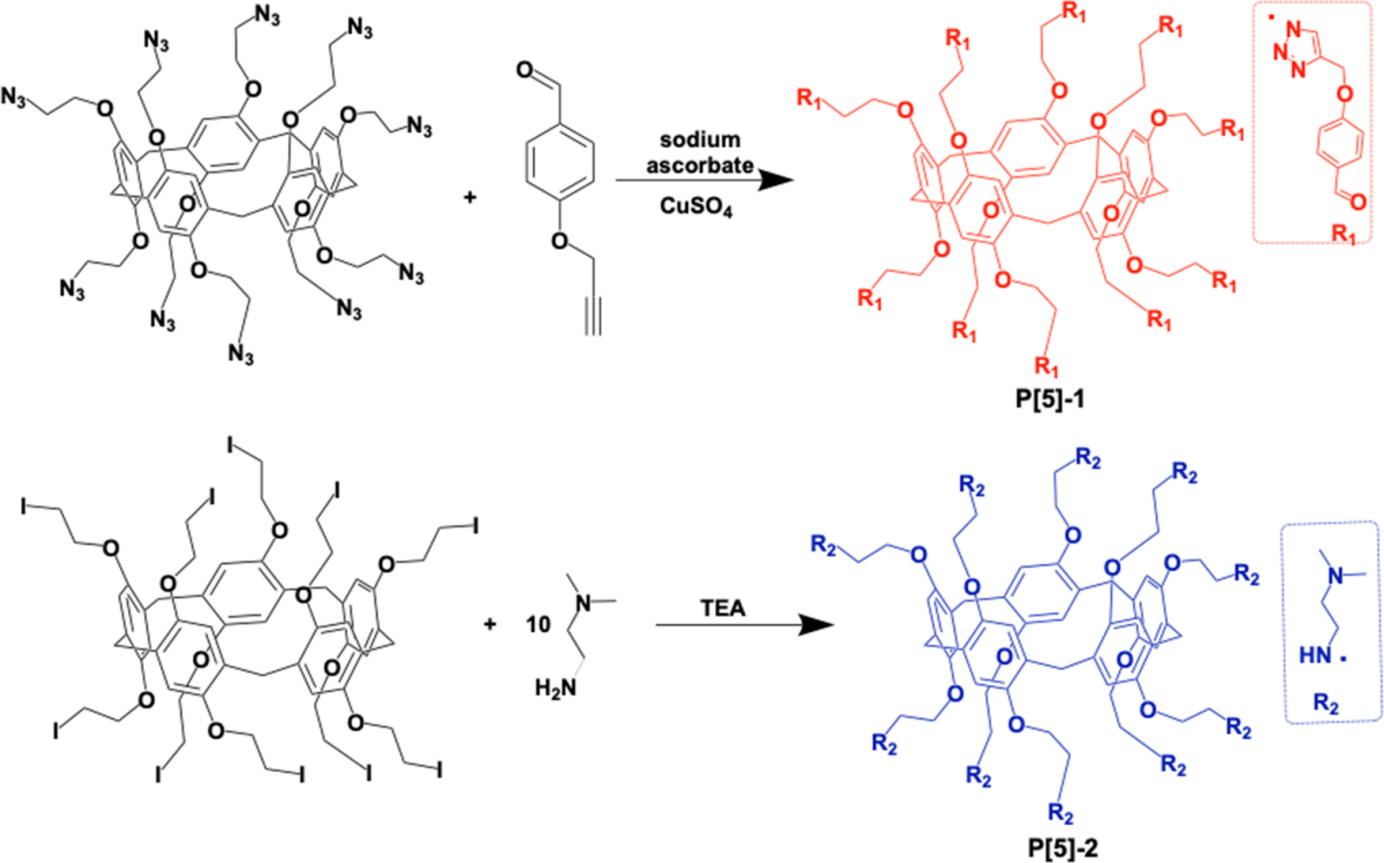
Synthesis Route of **P[5]-1** and **P[5]-2** Macrocycles

All compounds were prepared following previously reported methods^39,40^ (Scheme 1). To prepare target compounds, **P[5]-1** and **P[5]-2** were obtained from pillar[5]arene, including iodines or azides and 4-hydroxybenzaldehyde or *N*,*N*-dimethylethylenediamine. Carbon tetraiodide, carbon tetrabromide, boron trifluoride ethyl etherate, *N*,*N*-dimethylethylenediamine, triphenylphosphine, copper(II)sulfate, 4-hydroxybenzaldehyde, sodium ascorbate, triethylamine, paraformaldehyde, sodium hydride, and other solvent reagents were purchased from Alfa-Aeser, TCI Chemicals, Merck, and Sigma-Aldrich and used without further purification. ^1^H–^13^C NMR, Fourier transform infrared (FT-IR) spectroscopy, and elemental analysis of the compounds were carried out on a Varian 400 instrument (stand. TMS) at 298 K in CDCl_3_, a Bruker Fourier transform infrared (ATR), and a Leco CHNS 932, respectively (Figures S1–S12). All compounds were prepared using the synthesis method in the literature.^39^

### 2.2 Preparation of the P[5]-Based Spun Films

The spin coating method is based on the principle of dropping the solution onto the substrate with a microliter syringe or other apparatus and rotating it horizontally around an axis at a constant speed, while the solution spreads on the carrier surface under the effect of centrifugal force.

**Scheme 2:**
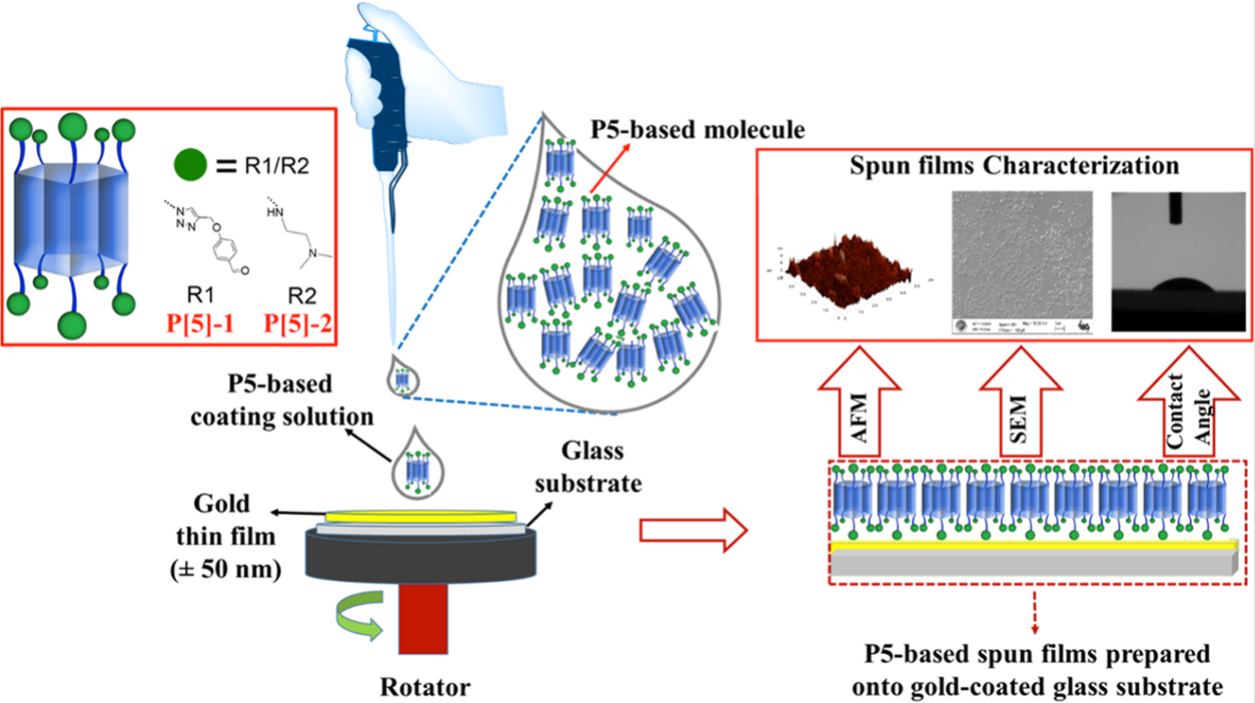
Schematic Presentation of the Preparation and Characterization of P[5]-Based Thin Films

The preparation of the P[5]-based thin films with the spin coating method, which is the method used in this work, is carried out via an SCS G3P-8 spin coating device. During the thin film preparation, the thin film preparation system was isolated with a glass cover against dust that could interfere with the film structure. Thanks to the control panel connected to the system, the rotation time and rotation speed can be adjusted. The pillar[5]arene/chloroform solutions were spread onto the rotating substrate using a microliter syringe. All thin films were produced at a rotational speed of 1000 rpm using 100 μL of these solutions at a concentration of 2.06 mg/mL. While the thin film was being formed, the rotating table was rotating at a constant rotation speed for 30 s by reaching the desired speed value in 7 s and stopping by slowing again in 7 s, and desiccation of the thin films occurred in 30 s under the same conditions. The fabricated P5-based thin films were used for scanning electron microscopy (SEM), contact angle, atomic force microscopy (AFM), and SPR measurements. Scheme 2 shows a schematic presentation for preparing P[5]-based thin films via the spin coating technique. Our spin coating system does not cover the thickness control during the fabrication process and it is well-known that the final film thickness of a spin coating film depends on the rotation speed. In this study, the thicknesses of the P[5]-based thin-film sensors are estimated using SPR curve data and WINSPALL fitting software (written by Wolfgang Knoll, developed at the Max-Plank Institute for Polymer Research, Germany).^41,42^

### 2.3 Surface Plasmon Resonance (SPR) Technique

The SPR system is based on the principle of energy transfer between metal electrons on a well-conducting metal-coated surface (gold, silver, copper, etc.) and electromagnetic waves sent to the surface. When energy transfer occurs between the metal surface and transmitted electromagnetic waves, a change in the intensity of the light reflected from the underside of the metal surface occurs. Surface plasmon resonance is determined when the change in the light intensity is measured. When light is sent at different angles to the metal surface, some of the light is reflected, and some is absorbed. This angle, which depends on the intensity of the reflected light, is called the resonance angle.

**Scheme 3:**
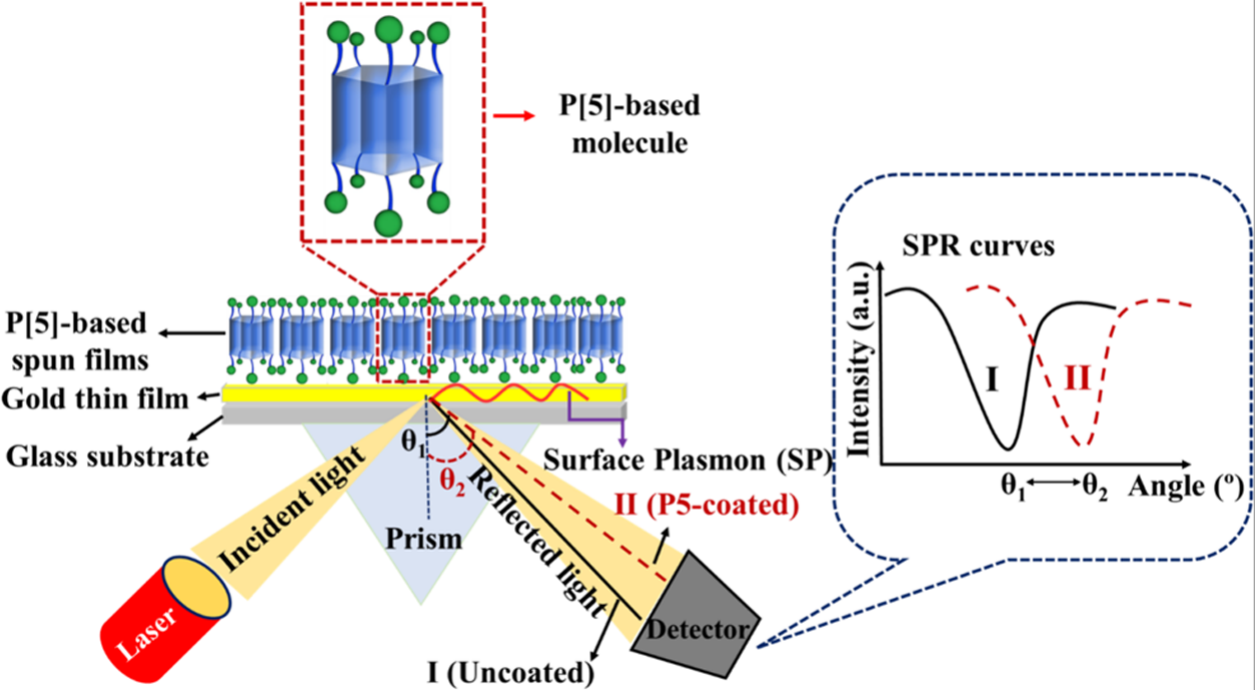
Schematic Representation of the SPR System

To generate surface plasmons during SPR measurements, a prism-based SPR system known as the Kretschmann configuration system was used. In this study, all gas sensing measurements were performed using a Biosuplar 6 Model SPR setup with a low-power HeNe laser (630–670 nm) light source and an angular resolution of about 0.003 degrees. A glass prism (*n* = 1.62) mounted onto a holder is available for measurement in air. 50 nm thin gold layer-coated glass slides supplied by TEKNOTIP company in Turkey had the dimensions of 20 × 20 mm, and the total thickness was 1 mm. All configurations are designed to directly, simultaneously, and without any labeling process measure the change in refractive index at the sensor surface. A schematic representation of the SPR system used in this study is shown in Scheme 3.

A liquid of the same index as the glass is applied between the glass and prism to ensure optical coupling. When the polarized beam encounters the gold film and the thin film interface on the surface of the gold film, total internal reflection occurs. In this case, as long as the angle of incidence is greater than the critical angle, waves of decreasing amplitude continue to be generated. Usually, the intensity of the reflected light does not change with the angle of incidence under the condition of total internal reflection. At an angle greater than the critical angle, the incident wave excites the delocalized electrons of the gold film, bringing them into resonance. In this state, maximum energy is absorbed, and surface plasmon resonance occurs. The intensity of the reflected light decreases sharply at this point, and the angle of incidence at which minimum reflection is observed is called the resonance angle or the SPR (θ) angle.

Dichloromethane, chloroform, ethyl acetate, carbon tetrachloride (99%, Sigma-Aldrich), trichloroethylene (99%, Thermo Scientific), and formic acid (85%, Isolab Chemicals) vapors were used without further purification as organic vapors for the **P[5]-1** and **P[5]-2** spun thin film sensor performances. The concentration values of each organic vapor in ppm can be calculated by the formula as follows:^43^
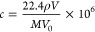
1where *c* (ppm) is the concentration value of vapor, ρ (g/mL) is the density of vapor, *V* (mL) is the volume, *M* (g/mol) is the vapor molecular weight, and *V*_0_ is the volume of the gas cell (∼0.02 mL). Using eq 1, the saturated concentrations of the vapors were calculated as 17494, 13970, 11466, 12477, 29688, and 11533 ppm for dichloromethane, chloroform, ethyl acetate, trichloroethylene, formic acid, and carbon tetrachloride vapors, respectively.

A special flow cell from transparent plastic, compatible for vapor measurements, was constructed to study the real-time SPR kinetic responses of **P[5]-1** and **P[5]-2** spun thin film sensors on exposure to selected vapor by measuring the SPR reflected light intensity changes as a function of time. The cell has two channels, with an inlet and outlet connected to silicone tubes. The inlet part is suitable for an injection of vapor into the gas cell. VOC injections were performed using a 1 mL syringe. 25% of this syringe was filled with saturated vapor and the rest of the syringe was filled with dry air in the first step of the kinetic response. The reciprocal exposures were applied by 50 and 75% of the saturated vapor, where the rest of the syringe was filled with dry air. The last step of the measurement was made with 100% saturated vapor. SPR kinetic measurements were recorded in real time for the laboratory conditions at room temperature with a relative humidity (RH) value of 25%. RH was controlled by mixing the dry air with wet air containing saturated vapor at 20 °C and was monitored by a HTC-2 LCD Digital Thermometer Hygrometer.

The SPR measurement system was completely controlled by Biosuplar Software, which handles the settings, the measurements for SPR curve or real-time SPR kinetics, and data acquisition as well as controlling the measurement and data presentation. The **P[5]-1** and **P[5]-2** spun thin film sensor responses were recorded as a function of time when they were periodically exposed to the organic vapors for at least 2 min and were then allowed to recover after injection of dry air. This procedure was carried out during several cycles to study the concentration changes at the ratios of 25, 50, 75, and 100% for each vapor. A constant concentration value (100%) was selected to observe the reproducibility of the **P[5]-1** and **P[5]-2** spun thin film sensors against dichloromethane vapor with three SPR kinetic measurements. All SPR kinetic results are discussed in Section 3.3.

### 2.4 Artificial Neural Networks (ANN)

**Scheme 4:**
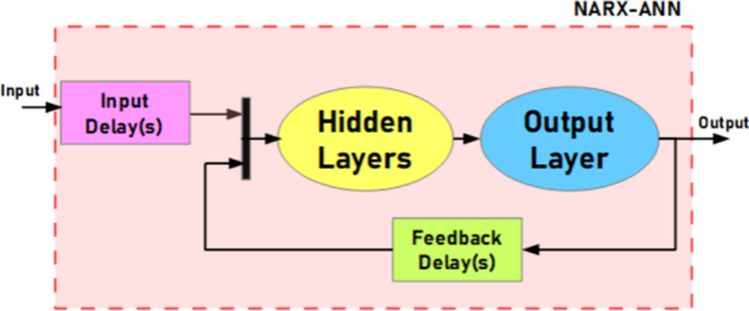
General Diagram of the ANN

Artificial neural networks (ANNs) are a class of machine learning models inspired by the structure and function of the human brain. They are composed of interconnected nodes or neurons organized into layers. ANNs typically consist of three types of layers: the input layer, where data are introduced into the network; one or more hidden layers, where complex transformations are applied to the input data; and the output layer, where the final predictions or classifications are generated. Neurons within the network are characterized by weights and bias terms, which are adjusted during the training process to minimize the difference between the network’s predictions and the true labels in the training data. By iteratively updating the weights and biases, the network learns to approximate complex relationships within the data, effectively creating a mathematical model that can generalize to unseen examples. The trained ANN can then be used for various tasks, such as classification, regression, and pattern recognition. Artificial neural networks are widely used in mathematical modeling or approximation of linear/nonlinear processes. ANNs have a few types of structures that are selected by the designer. In this work, NARX-ANN input was designed to model normalized reflected light intensity values. A general diagram of the NARX-ANN is given in Scheme 4.

The proposed NARX-ANN scheme is trained with the Levenberg–Marquardt algorithm using an MSE performance function. Our data set comprises measurements taken at 1 s intervals over a 120 s period, resulting in a total of 120 measurements to be modeled. To ensure robust training and evaluation of the NARX-ANN model, we employed a data partitioning strategy. Specifically, 70% of the experimental data, equivalent to 84 measurements, is randomly selected for training. Subsequently, 15% of the data, corresponding to 18 measurements, is reserved for validation purposes, while another 15% (also 18 measurements) is set aside for testing the NARX-ANN model. This partitioning scheme enables us to effectively assess the model’s performance while maintaining sufficient data for training, validation, and testing phases.

## 3 Results and Discussion

### 3.1 Characterization of the Compounds

**Figure 1:**
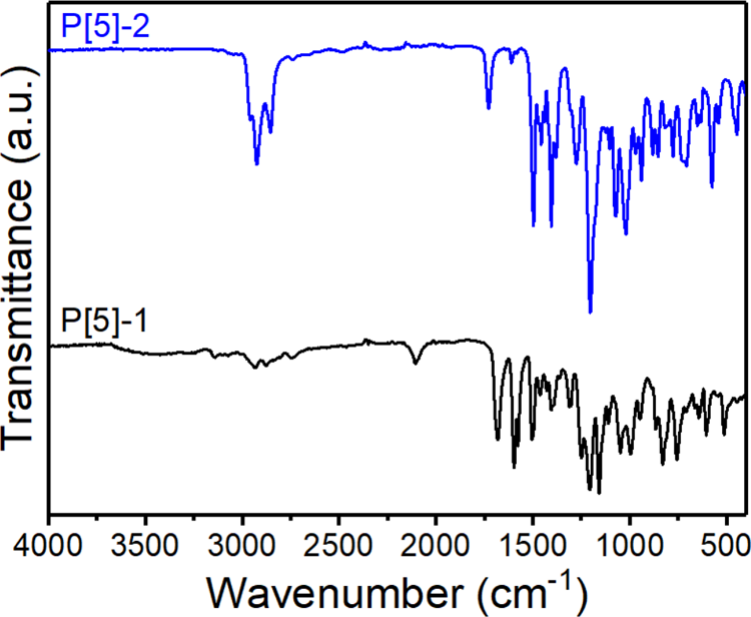
FT-IR spectra of **P[5]-1** and **P[5]-2**.

FT-IR spectra were performed on the functional groups of the target compounds and the reagents (Figures 1 and S1–S4). **P[5]-1** presents a clear spectrum that the broad peak at 1202 cm^–1^ was assigned to the C–O stretching of the etheric fragments on the main pillar[5]arene skeleton. The C=O vibrations of the aldehyde units were observed at 1728 cm^–1^, while multi–C–H stretching appeared at 2822, 2924, and 3009 cm^–1^ in small peaks. Moreover, multiple peaks at 1496, 1455, and 1404 cm^–1^ indicate the C=C bonds of the aromatic groups in the structure. On the other hand, in the FT-IR spectrum of **P[5]-2**, the weak peaks at 2963, 2930, and 2839 cm^–1^ was attributed to the C–H stretching of the alkyl chains and the conjugative moieties. The broadest peak of the spectrum assigned to the C–O stretching vibrations was observed at 1202 cm^–1^. The carbon–carbon double bond vibrations of the aromatic fragments appeared at 1497, 1456, and 1405 cm^–1^. The medium peak at 1611 cm^–1^ was attributed to the bending vibration of the C–N bonds.

To characterize the structure of the reagents and target macrorings, NMR spectroscopy was performed using ^13^C and ^1^H NMR spectra (Figures S5–S12). In the ^1^H NMR spectrum of **P[5]-1**, the bridge CH_2_ protons on the main skeleton were observed at 3.77 ppm. The protons in the triazole rings were observed around 7.80 ppm in overlapping form with other benzene units, whereas the aldehyde protons were observed at 9.95 ppm. Three different CH_2_ protons on **P[5]-1** appeared at 5.10, 4.78, and 4.30 ppm in singlet, triplet, and triplet forms, respectively. In the ^1^H NMR spectrum of **P[5]-2**, the bridge CH_2_ protons on the main skeleton were observed at approximately 3.80 ppm as multiplet signals in an overlapping form with other CH_2_ protons. The CH_3_ protons bound to nitrogen of the amine fragments were observed at 2.04 ppm in singlet form, whereas other different CH_2_ protons appeared at 3.33, 2.45, and 2.52 ppm in the singlet, triplet, and triplet forms, respectively. The broad and small peak at 4.25 assigned hydrogens to the NH moieties.

### 3.2 Characterization of the P[5]-Based Spun Films

**Figure 2:**
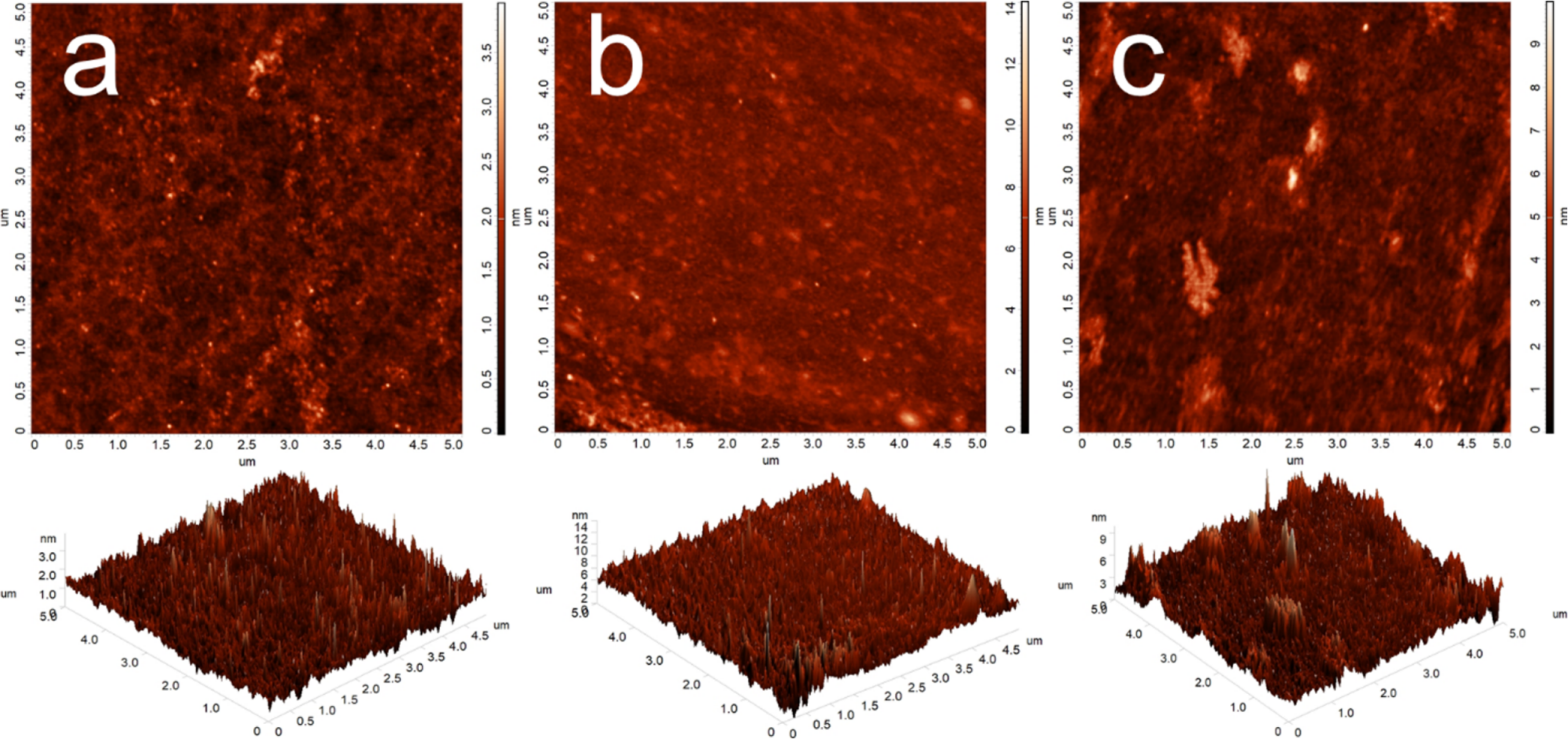
AFM images of bare glass (a), the **P[5]-1**-coated glass substrate (b), and the **P[5]-2**-coated glass substrate (c).

Figure 2 shows the AFM images of the pillar[5]arene derivatives, revealing the nanometric structures of the films. The films are quite homogeneous and smooth; however, the film for **P[5]-1** is slightly more uniform because the number of islands is much greater (Figure 2b). As can be clearly seen in Figure 2, it is obtained on a 5 × 5 μm scale that the area roughness and root-mean-square (rms) are 0.23 and 0.31 nm for the bare glass substrate, 0.61 and 0.85 nm for the **P[5]-1** film, 0.63 and 0.89 nm for the **P[5]-2** film, respectively. In addition, the SEM images showed that the morphologies of the **P[5]-1** and **P[5]-**2-coated surfaces were different, and the element contents of the surfaces were analyzed by energy-dispersive X-ray spectroscopy (EDX) (Figure S13). The wettability of the surfaces was also examined by contact angle measurements, and the water contact angles of the bare glass, **P[5]-**1-coated, and **P[5]-**2-coated surfaces were found to be 5.12 ± 0.89, 26.23 ± 1.12, and 48.89 ± 1.65°, respectively (Figure S14). The fact that there are more groups that increase the hydrophilicity in the structure of the **P[5]-1** molecule confirms these results.

### 3.3 SPR Gas Measurements

Thickness values of the P[5]-1 and P[5]-2 thin-film sensors were calculated via WINSPALL fitting program and found to be 7.6 nm for **P[5]-1** and 5.9 nm for **P[5]-2** thin films, respectively. WINSPALL fitting graphs for both sensors are given in Figures S15–S16. The interaction mechanism of organic vapors with the thin-film sensor occurs in three steps: adsorption to the surface, diffusion, and desorption. When a **P[5]-1** thin-film sensor coated on a gold-coated glass surface is exposed to organic vapor, the vapor molecules physically attach to the thin film surface. Because of the adsorption effect of the film surface, a rapid change in the reflected light intensity value is observed, and this interaction, called the fast process, continues with the diffusion of vapor molecules attached to the surface into the thin film. With the increase in the number of diffusing molecules, swelling of the thin-film sensor occurs, and accordingly, it will be difficult for vapor molecules to diffuse into the thin film. This interaction is referred to as a slow process. The interaction mechanism ends when the vapor molecules are attached to the surface or the diffusing vapor molecules leave the surface. When air is introduced into the environment, a rapid decrease in the light intensity is observed because of the desorption of organic vapor molecules attached to the surface.

**Figure 3:**
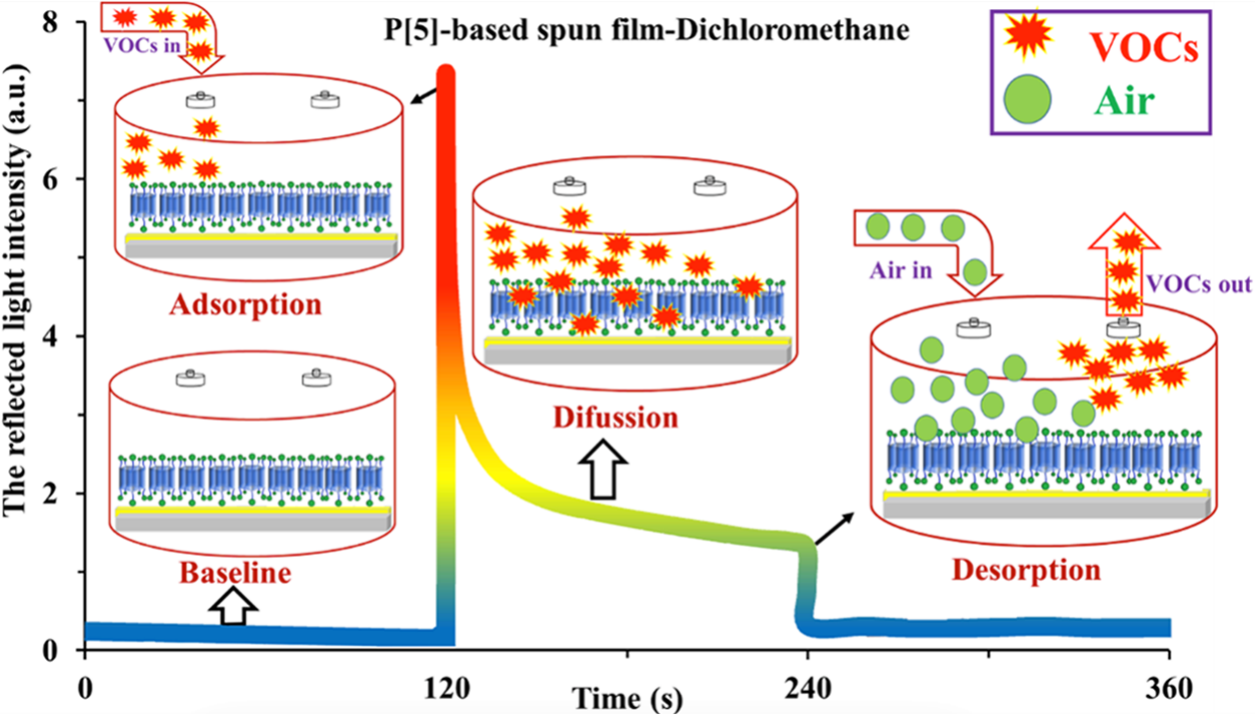
Interaction of a **P[5]-1**-based spun thin film with dichloromethane vapor.

The interaction of the **P[5]-1**-based spun thin film with dichloromethane vapor is shown in Figure 3. During the first 120 s, fresh air is in the environment. At 120 s, when dichloromethane vapor is introduced into the environment at a saturated concentration, the reflected light intensity increases rapidly. This rapid change in the light intensity indicates that the thin film interacts rapidly with the dichloromethane vapor. The dichloromethane vapor remained in the environment for 2 min, and during this period, the reflected light intensity showed a certain change and then approached a constant value. At 240 s, after air was introduced into the environment, the reflected light intensity returned to approximately its initial value. This result shows that the reversibility of the thin-film sensor is very high.

**Figure 4:**
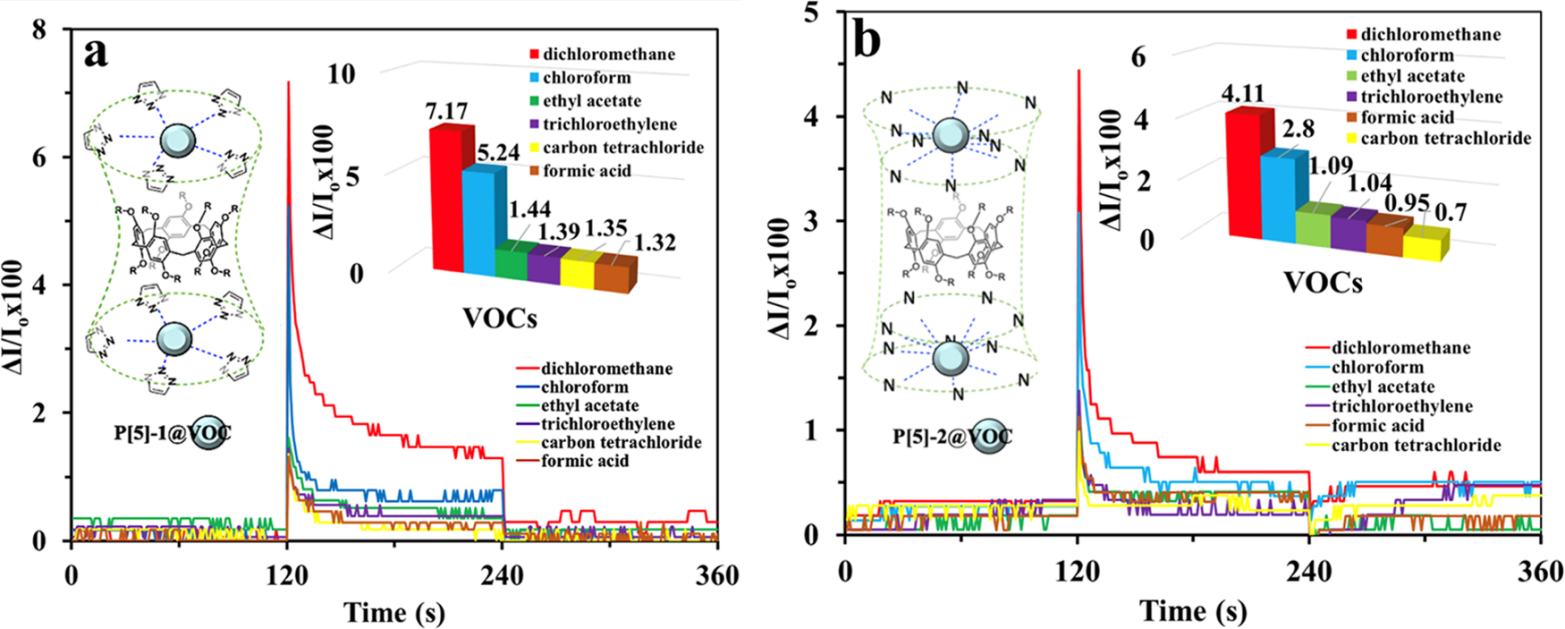
Kinetic response of (a) **P[5]-1** and (b) **P[5]-2** thin-film sensors to different VOCs. Insets: the representation of the interaction between the macrorings and VOCs.

The interaction of the prepared **P[5]-1** and **P[5]-2** spun thin film sensors with six different VOCs was investigated, and the response of the spun thin film sensors to these VOCs is shown in Figure 4.

The difference in the reflected light intensity before the interaction between the thin film and VOC molecules and after exposure to selected VOC is expressed as Δ*I*. Here, it can be written as Δ*I* = *I* – *I*_o_, where *I*_o_ and *I* are the reflected light intensities at the initial and binding moment, respectively. The harmful vapor response in kinetic studies of thin films is defined as
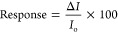
2

Figure 4 displays a set of typical curves showing the reflection intensity response as a function of time when the **P[5]-1** and **P[5]-2** spun thin film sensors are exposed to saturated vapors for 2 min. The initial stage in SPR kinetic measurements is to flush dry air as a reference gas through **P[5]-1** and **P[5]-2** spun thin film sensors to obtain a baseline (*I*_0_) from 0 to 120 s. From 120 to 240 s, the **P[5]-1** and **P[5]-2** spun thin film sensors are exposed to the selected organic vapor. This causes a change in the reflected light intensity until the sensor reaches a steady state (*I*). After an injection of dry air into the gas cell, the vapor molecules are released from the **P[5]-1** and **P[5]-2** spun thin film sensors. Moreover, the sensor response returns back to its baseline depending on the number of releasing vapor molecules. These processes are clearly shown in Figure 3 for the interaction of the **P[5]-1**-based spun thin film with dichloromethane vapor. As seen in Figure 4, all organic vapors yielded a rapid response in a few seconds during 120 s, and then the reflected light intensity continued to decrease. As dry air was ejected into the gas cell in 240 s, the interacting organic vapor molecules with the **P[5]-1** and **P[5]-2** spun thin film sensors moved away from the gas cell and tried to return to their initial state. While chloroform and dichloromethane vapors completely recovered, other vapors did not fully return to the baseline state. In this case, incompletely returnable vapors have been neglected because of their low response and bad recovery. When evaluated in terms of selectivity, the **P[5]-1** and **P[5]-2** spun thin film sensors give the highest responses to dichloromethane and chloroform vapors. This result can be concluded that both materials are more selective to dichloromethane and chloroform vapors than the others. In addition, the response of the **P[5]-1** spun thin film sensors is higher than the response of the **P[5]-2** spun thin film sensors. Furthermore, the efficiency in the detection of volatile organic compounds increased due to more intermolecular interactions of multinucleophilic groups (nitrogens, aromatic rings, and oxygens) on the target compounds.^44,45^ Both target molecules have a large number of donor atoms, providing them with an electronic cage to trap VOCs. Potential interactions between macrocycles and VOCs are schematized in the inset of Figure 4.

Figure 4 and Table 1 show that P[5]-based organic materials, whose chemical sensor properties were investigated, show high responses to dichloromethane and chloroform vapors. However, such a behavior of the sensors can be explained by the interaction between the chemical structure of the material and the organic vapor. It is well-known that the H-bonding ability, π–π interaction, host–guest interaction, and physical parameters of VOCs play an important role in the vapor sensing process between organic thin film materials and VOC vapors.^46−48^ Because the electronic cage of **P[5]-1** is more appropriate, it easily captures the VOC molecules with the molecular interaction principle. In particular, the nitrogens on the multitriazole units enable an excellent hydrogen bonding interaction between macroring and VOCs.^49−53^ When the diameter of pillar[5]arene after bounded triazole fragments is compared with the diameter of the pillar[5]arene, including azide moieties, **P[5]-1** has a larger cavity that is ideally suited for larger solvents. Even though it is not as good, owing to the long chains, **P[5]-2** interacted with the studied solvents depending on the intermolecular interaction or hydrogen bonding. A pillar[5]arene-quinoline Langmuir–Blodgett (LB) film was used for the detection of organic vapors, such as benzene, toluene, dichloromethane, and chloroform. The highest responses were observed for dichloromethane and chloroform vapors due to the appropriate cavity and host–guest inclusion character of the pillar[5]arene-quinoline material.^5^

**Table 1:** Physical Properties of VOCs and Response Values of P[5]-Based Sensors

VOCs	dipole moment (*D*)	molar volume (cm^3^ mol^–1^)	vapor pressure (kPa, 25 °C)	**P[5]-1** response (Δ*I*/*I*_o_ × 100)	**P[5]-2** response (Δ*I*/*I*_o_ × 100)
dichloromethane	1.60	64.10	46.53	7.17	4.11
chloroform	1.08	80.70	26.66	5.24	2.80
carbon tetrachloride	0	97.10	15.06	1.44	1.09
ethyl acetate	1.78	97.80	12.13	1.39	1.04
trichloroethylene	0.80	89.70	7.73	1.35	0.95
formic acid	1.41	37.80	4.53	1.32	0.70

The **P[5]-1** and **P[5]-2** thin-film sensors showed higher responses to dichloromethane vapor compared to other organic vapors. It was observed in the experiments that these thin-film sensors have selectivity toward dichloromethane vapor. The results of the interaction between P[5]-based thin films and VOCs can be explained in terms of the physical parameters of VOCs. Because the dichloromethane molecule has a large dipole moment, low molar volume, and high vapor pressure, it is thought that these vapor molecules will diffuse faster and more easily into P[5]-based thin films. Although the dipole moment of the ethyl acetate molecule has the highest value compared with other VOC molecules, its Δ*I*/*I*_o_ × 100 value is lower. This vapor molecule is predicted to diffuse slower and with more difficulty into P[5]-based thin films due to its low vapor pressure and highest molar volume compared with the others. A similar relationship can be established for the response results of thin-film sensors to formic acid. Although the formic acid molecule has the lowest molar volume, it is thought to be more difficult to diffuse into P[5]-based thin films because of the lowest vapor pressure value.

**Figure 5:**
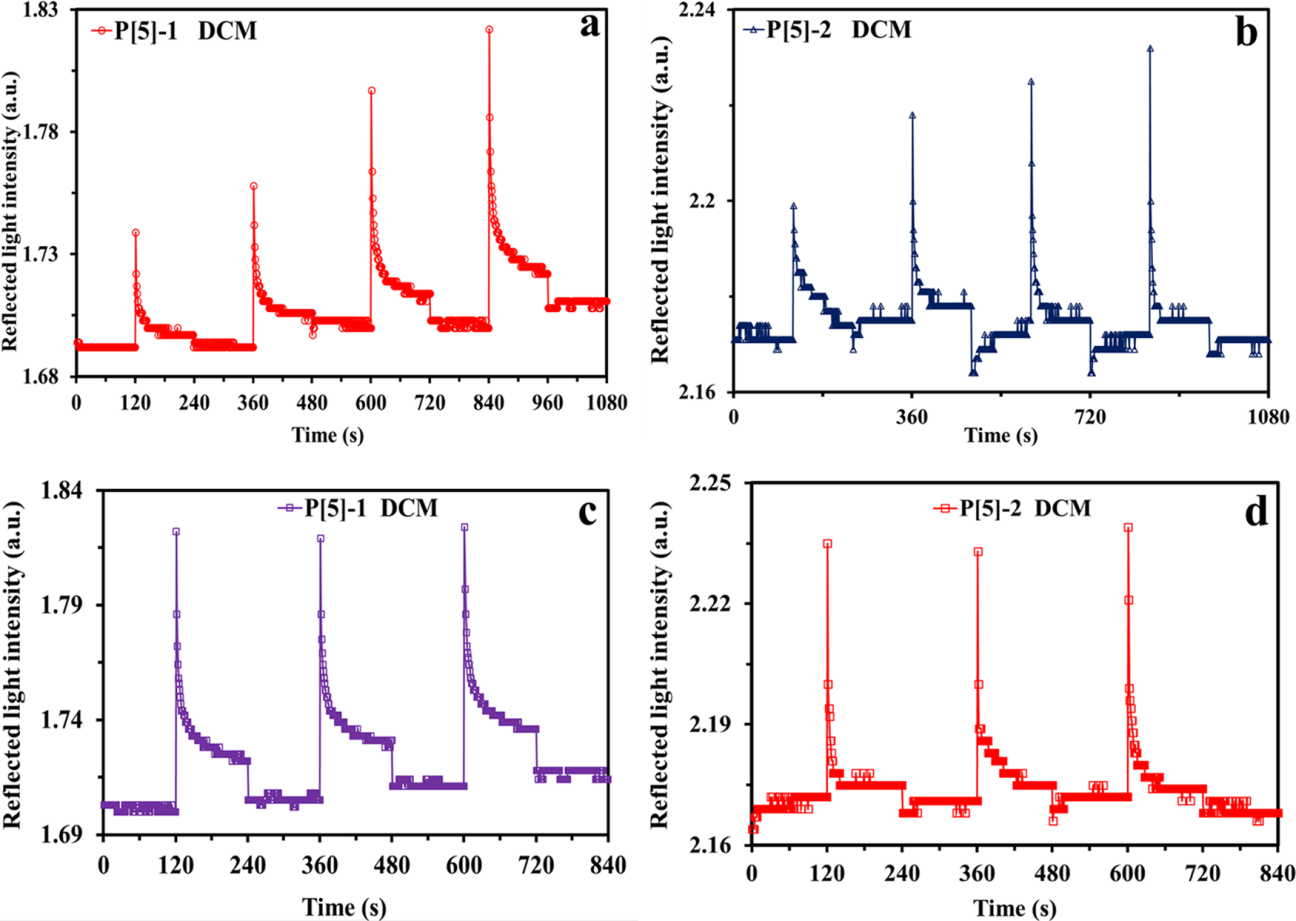
Response of **P[5]-1** and **P[5]-2** thin-film sensors to dichloromethane vapor at different concentrations (25, 50, 75, and 100%) (a, b) and (b), three cycles at 100% concentrations (c) and (d), respectively.

It is important to study the concentration effect of any sensor material to calculate the sensitivity (*S*), limit of detection (LOD), and limit of quantification (LOQ) values. Therefore, 25, 50, 75, and 100% concentration values of each vapor are exposed to the **P[5]-1** and **P[5]-2** spun thin film sensors to investigate the concentration dependence of the materials. In addition, the reproducibility of a sensor material means the sensor material can produce the same results when used repeatedly under the same concentration values. Figure 5 shows the dichloromethane vapor responses of the **P[5]-1** and **P[5]-2** spun thin film sensors (i) at 4 different concentration values (for the determination of *S*, LOD, LOQ parameters) and (ii) at a constant concentration value (for reproducibility test).

The dependence of concentration responses for the **P[5]-1** and **P[5]-2** spun thin film sensors is given in Figure 5a,b. The **P[5]-1** spun thin film sensor shows more stability than the **P[5]-2** spun thin film sensor with similar responses. As the concentration of dichloromethane vapor increases, the reflected light intensity of the **P[5]-1** spun thin film sensor response also increases. It is clearly seen that the **P[5]-2** spun thin film sensor shows lower responses with an unstable behavior when we compare with the **P[5]-1** spun thin film sensor. As seen in Figure 5c,d, the response of the **P[5]-1** spun thin film sensor at a constant concentration value is similar, reversible, and reproducible. The responses in the **P[5]-2** spun thin film sensor are smaller and unstable. These results indicated that the **P[5]-1** spun thin film sensor has better performance.

It is clear from the existing literature that there are very few similar studies using pillar[5]arene-based materials as sensing elements and testing gas measurements with QCM or SPR techniques. The sensitivity of pillar[5]arene-based thin-film chemical sensors to harmful organic vapors has been investigated using gas measurement techniques (QCM and SPR).^5,6,40,54,55^ In these studies, P[5]-biphenylcarboxylic acid (P5-BPCA),^6^ P[5]arene-quinoline (P5-Q),^5^ P[5]arene-2-amino-3-hydroxypyridine (P5-AP),^40^ P[5]arene-salicylaldehyde (P5-S),^55^ and P[5]arene-deca pyridin-2-amine bearing (P5-PA)^54^ materials were preferred as both thin film and sensing element materials. The kinetic measurement results of these P[5]-based thin-film gas sensors in the literature are similar to the results of the present study, and it is observed that P[5]-based thin-film sensors show a higher response and sensitivity to dichloromethane (DCM) and chloroform vapors than other harmful vapors.

**Figure 6:**
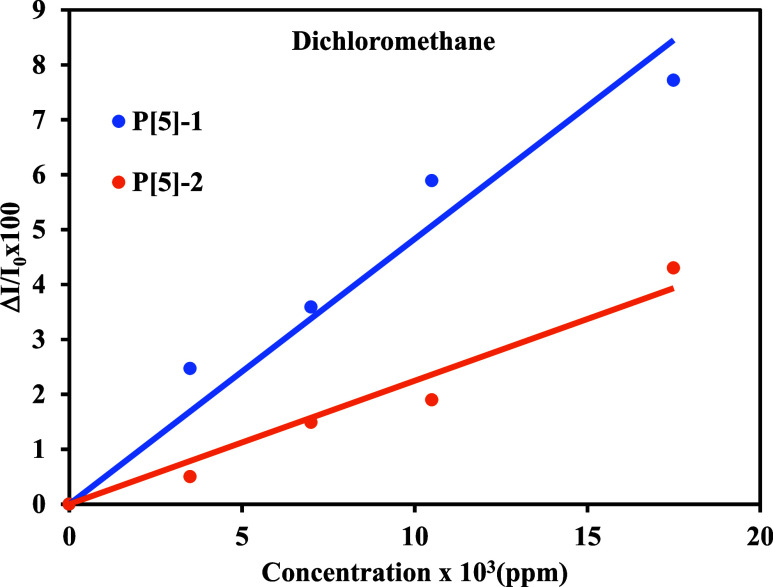
Calibration curves of the **P[5]-1** and **P[5]-2** thin-film sensors with regard to dichloromethane vapor at increasing concentrations.

To calculate the *S*, LOD, and LOQ values, calibration curves of both P[5]-based sensors as a result of the exposure to increasing concentrations of the dichloromethane vapor are plotted using the kinetic data and are given in Figure 6. Calibration curves in exposure to chloroform, trichloroethylene, carbon tetrachloride, ethyl acetate, and formic acid were generated, and the below-mentioned calculations of the sensor parameters were performed using these calibration curves (given Figures S17–S21). Taking advantage of the linear dependency between the response values with the increasing concentration of the VOCs plotted, sensitivities of the **P[5]-1-** and **P[5]-**2-based SPR sensors were calculated. The slope of the linear correlation gives the sensitivity (*S*); the responses are ppm value of the VOC.

The LOD value, which describes the lowest concentration value that can be reliably detected, is given by eq 3:^56^
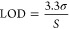
3

The LOQ value which is the lowest concentration value that can be reliably quantified accurately is given by eq 4:^57^
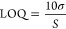
4

Both limits depend on the *S* value of the P[5]-based sensor and the standard deviation of the SPR instrument. The *S* value obtained using the calibration curves and the standard deviation of σ = 0.001 were used to determine the LOD and LOQ values. All parameters including the correlation coefficient (*R*^2^), which is close to unity, are presented in Table 2. The highest sensitivity and therefore the lowest LOD and LOQ values were observed for dichloromethane vapor. LOD and LOQ values were found to be 6.83 and 20.69 ppm, respectively. This result is in accordance with the highest response value indicated in Figure 4. The compared values of the sensor parameters also showed that the **P[5]-1** thin-film sensor showed higher sensitivity and hence lower LOD and LOQ values with respect to the **P[5]-2** thin-film sensor.

**Table 2:** Sensor Parameters

P[5]-based sensor	VOCs	sensitivity (response/ppm) × 10^–4^	LOD (ppm)	LOQ (ppm)	*R*^2^
**P[5]-1**	dichloromethane	4.83	6.83	20.69	0.984
	chloroform	3.91	8.45	25.59	0.975
	ethyl acetate	2.83	11.65	35.31	0.960
	trichloroethylene	2.09	15.74	47.71	0.971
	carbon tetrachloride	1.91	17.25	52.27	0.983
	formic acid	1.42	23.16	70.17	0.999
**P[5]-2**	dichloromethane	2.25	14.68	44.48	0.982
	chloroform	2.93	11.26	34.12	0.991
	ethyl acetate	1.60	20.65	62.58	0.968
	trichloroethylene	1.17	28.08	85.11	0.978
	carbon tetrachloride	1.51	21.91	66.40	0.987
	formic acid	0.53	62.26	188.68	0.980

### 3.4 ANN Modeling Results

**Figure 7:**
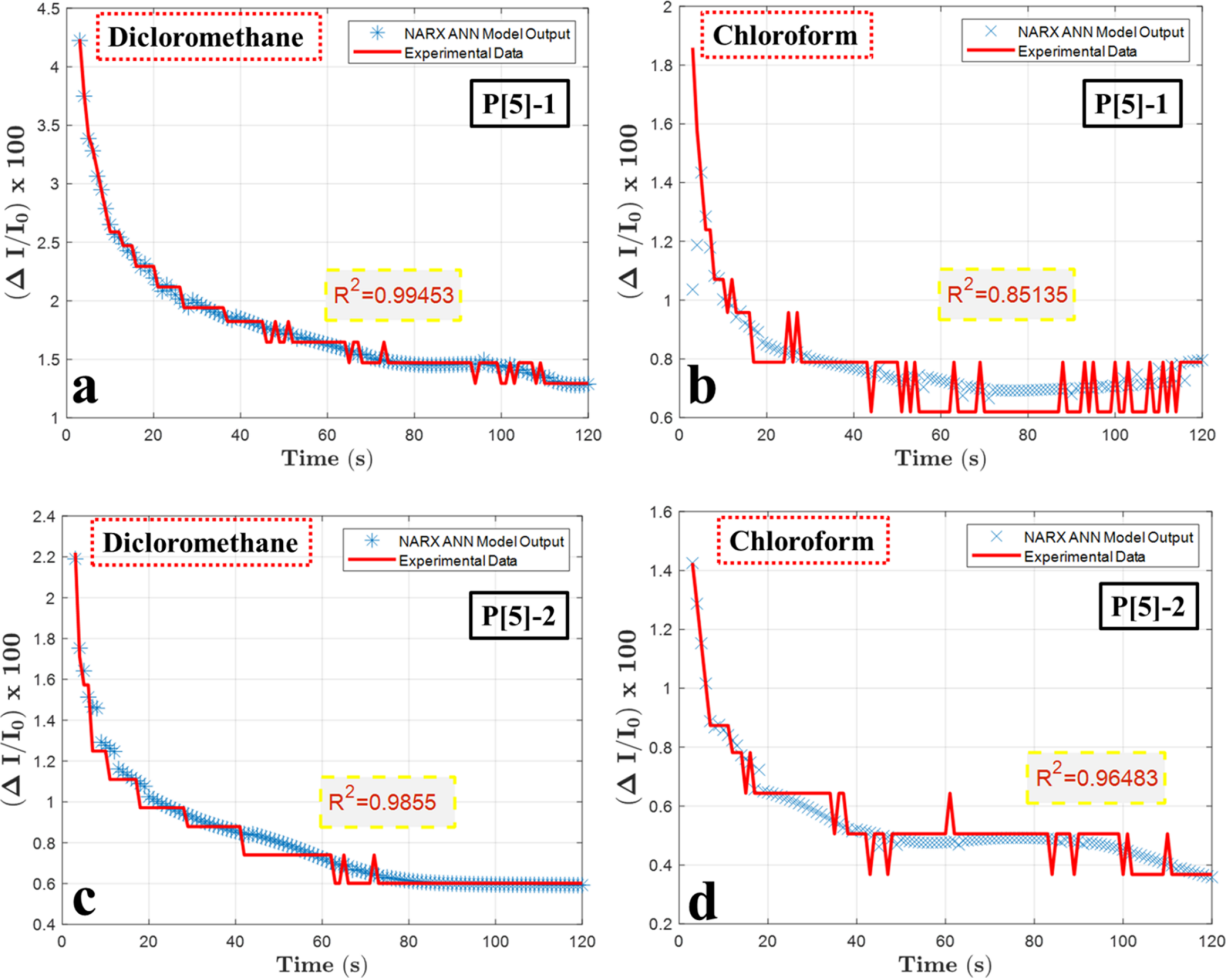
ANN modeling results of (a) dichloromethane-(**P[5]-1**), (b) chloroform vapors for **P[5]-1**, and (c) dichloromethane and (d) chloroform vapors for **P[5]-2** chemical sensors.

To validate the ANNs, NARX-ANN is trained and tested using experimental data. Figure 7 shows the NARX-ANN modeling results of normalized intensity values for dichloromethane and chloroform in both prepared **P[5]-1**- and **P[5]-**2-based SPR sensors. In this figure, the NARX-ANN modeling results of normalized intensity values during the interaction between the dichloromethane/chloroform and P[5]-based SPR sensors are shown. Similarly, the NARX-ANN modeling results of the normalized intensity values for other vapors tested in this work are given in Figures S22–S29.

Considering Figure 7a,c, the NARX-ANN dichloromethane model successfully converges to the experimental data. In both figures, the correlation coefficients correspond to 0.99 and 0.98, respectively. The modeling results for **P[5]-1** and **P[5]-2** for dichloromethane are satisfactory.

For the chloroform modeling results shown in Figure 7b,d, the correlation coefficients are 0.85 for **P[5]-1** and 0.96 for **P[5]-2**, respectively. Both results are acceptable, considering the correlation coefficients and performance metrics given in Table 3.

**Table 3:** ANN Modeling Error Performance Metrics

P[5]-based sensor	VOCs	MSE	variance	std. dev.
**P[5]-1**	dichloromethane	0.00301	0.003034	0.055083
	chloroform	0.012428	0.012148	0.110217
	ethyl acetate	0.002468	0.002428	0.049276
	trichloroethylene	0.001722	0.001664	0.040787
	carbon tetrachloride	0.001262	0.001242	0.035249
	formic acid	0.002679	0.00269	0.051862
**P[5]-2**	dichloromethane	0.002844	0.002308	0.048038
	chloroform	0.002223	0.002184	0.046735
	ethyl acetate	0.001613	0.001617	0.040215
	trichloroethylene	0.002343	0.002352	0.048495
	carbon tetrachloride	0.004459	0.004302	0.065587
	formic acid	0.000824	0.000827	0.028751

## 4 Conclusions

In the current work, two novel and well-characterized pillar[*n*]arene-based compounds (**P[5]-1** and **P[5]-2**) were prepared as SPR optical sensors to investigate their gas sensing abilities during exposure to six different VOCs. **P[5]-1-** and **P[5]-**2-based SPR optical sensors were fabricated onto gold-coated glass substrates via spin coating. To investigate the homogeneity of these films, AFM, SEM, and contact angle were used. The P[5]-based SPR sensors exhibited consistent and reproducible responses when exposed to both saturated and various concentrations of the tested VOCs. Notably, both P[5]-based SPR sensors exhibited the highest response to dichloromethane among the tested VOCs. The NARX-ANN model was trained and tested using SPR experimental data to validate the ANNs. The aim of this process is to compare the experimental data obtained from SPR gas measurements with the modeling results by analyzing their correlation coefficients and some performance metrics, such as the mean squared error (MSE) and standard deviations. The modeling results in the **P[5]-1** and **P[5]-2** SPR experimental data for dichloromethane vapor are considered satisfactory and acceptable, with the correlation coefficients corresponding to 0.99 and 0.98, respectively. In conclusion, the **P[5]-1-** and **P[5]-2**-based SPR sensors can be used as potential devices at room temperature for the early detection of dichloromethane vapor. In future work, new pillar[6]arene (P[6])-based macrocyclic compounds will be synthesized to investigate their thin film properties and gas sensing abilities by comparing the performances of the P[5]-based SPR sensors.

## Supporting Information Available

The synthesis of pillar[5]arene-based macrocyclic compounds; FT-IR spectra; H NMR spectra; C NMR spectra; SEM images; contact angle images; the fitting graphs for pillar[5]arene coated thin-film sensors; the calibration curves for pillar[5]arene coated thin-film sensors; ANN modeling results of pillar[5]arene coated thin-film sensors (PDF)
