# Supplementary material for: Sensing Volatile Pollutants with Spin-Coated Films Made of Pillar[5]arene Derivatives and Data Validation via Artificial Neural Networks
Source: ACS Appl Mater Interfaces. 2024 Jun 5;16(24):31851–63. doi: 10.1021/acsami.4c06970 (PMC11194768; doi:10.1021/acsami.4c06970)
Supplement: Supplementary file 1 — am4c06970_si_001.pdf [file am4c06970_si_001.pdf]

Supporting Information

Sensing Volatile Pollutants with Spin-Coated Films made of Pillar[5]arene Derivatives and Data Validation via Artificial Neural Networks

Ahmed Nuri Kursunlu[1,*‡], Yaser Acikbas[2,*‡], Ceren Yilmaz[1], Mustafa Ozmen[1], Inci Capan[3], Rifat Capan[3], Kemal Buyukkabasakal[4], Ahmet Senocak[5]

[*] Corresponding authors.

E-mail addresses: yaser.acikbas@usak.edu.tr (Y. Acikbas), ankursunlu@gmail.com (A.N. Kursunlu).

[‡] These authors contributed equally to this work.

[1] Department of Chemistry, Faculty of Science, University of Selcuk, 42250, Konya, Türkiye

[2] Department of Materials Science and Nanotechnology Engineering, Faculty of Engineering, University of Usak, 64200, Usak, Türkiye

[3] Department of Physics, Faculty of Science, University of Balikesir, 10145, Balikesir, Türkiye

[4] Department of Electrical and Electronics Engineering, Faculty of Engineering, University of Usak, 64200, Usak, Türkiye

[5] Department of Chemistry, Gebze Technical University, 41400 Gebze, Kocaeli, Türkiye

The synthesis of 4-(prop-2-yn-1-yloxy)benzaldehyde

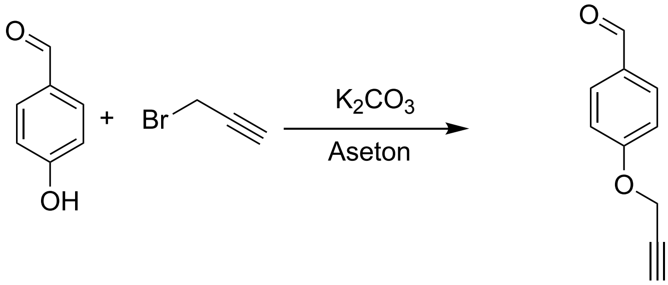

4-(prop-2-yn-1-yloxy)benzaldehyde was prepared as a known procedure [1]. 4-hydroxybenzaldehyde (20 mmol), potassium carbonate (60 mmol) and propargyl bromide (60 mmol) were dissolved in acetone (150 mL). The mixture was refluxed for overnight and cooled to room temperature. The mixture was extracted with dichloromethane/water and the collected organic phases. A yellow-white solid was obtained. M.P. 78 °C; ^{1}H-NMR ($CDCl_3$): 9.91 (s, 1H, CH=O), 7.86 (d, 2H, PhH), 7.09 (d, 2H, PhH), 4.79 (s, 2H, CH_2), 2.58 (s, 1H, CH).

The synthesis of pillar[5]arene including azides

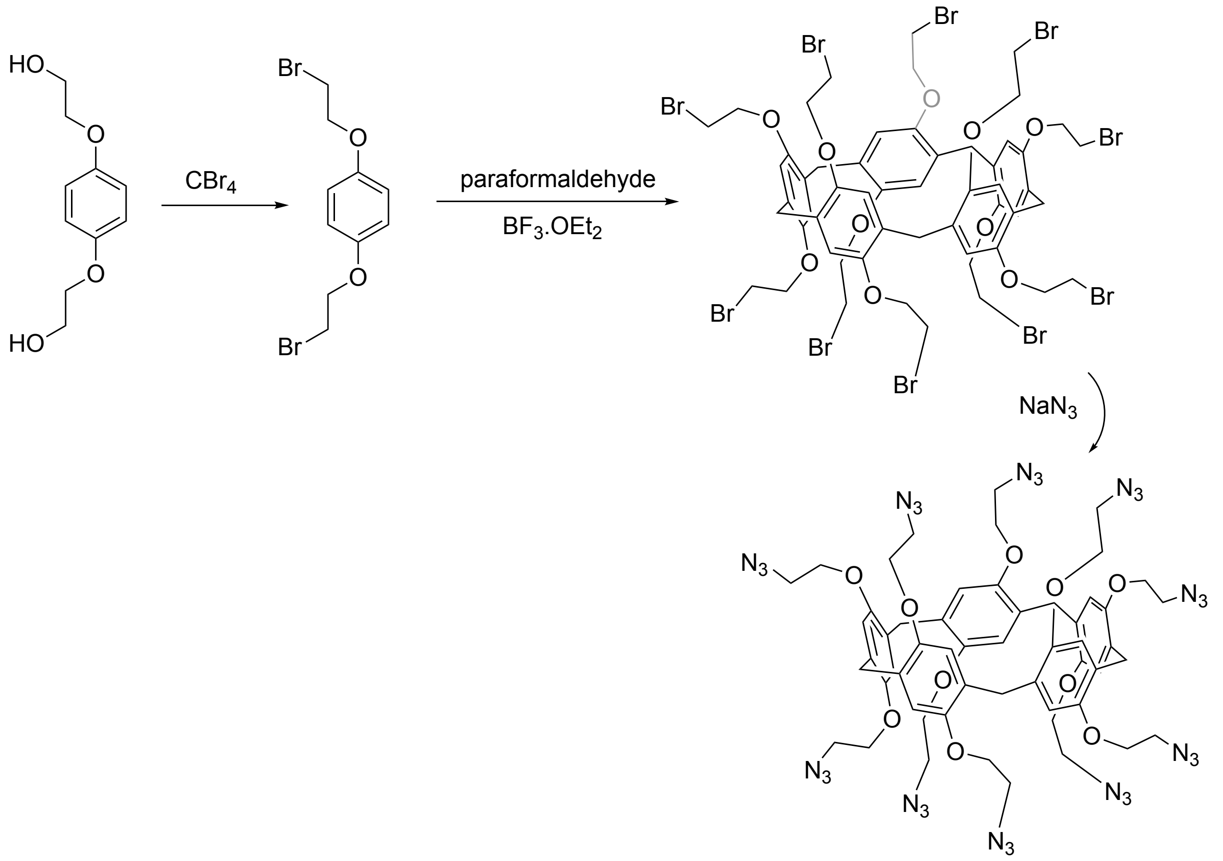

Pillar[5]arene including iodines was prepared according to a literature procedure [2]. Triphenylphosphine (30 mmol) was solved in a flask (150 mL dry acetonitrile) and poured 1,4-bis(2-hydroxyethoxy)benzene (125 mmol) to this solution and the reaction mixture was stirred at 0 °C for 1h. Then, carbon tetrabromide (60 mmol) was slowly added and the mixture was mixed for 7 h under nitrogen atmosphere. Following to the completion of reaction, ice pieces was added to the reaction flask. The obtained white product was washed with methanol/water mixture (50:50), and then re-crystallized from methanol (6.43 g, 81%). ^{1}H NMR (400 MHz, $CDCl_3$) δ (ppm): 3.65 (t, J = 6.3 Hz, 4H, CH_2), 4.28 (t, J = 6.3 Hz, 4H, CH_2), 6.91 (s, 4H, PhH). ^{13}C NMR (75 MHz, $CDCl_3$) δ (ppm): 152.5, 117.7, 69.1, 29.1.

Paraformaldehyde (17 mmol) was solved in 1,2-dichloroethane (100 mL), 1,4-bis(2-bromoethoxy)benzene (5.5 mmol) was added this solution. Boron trifluoride diethyl etherate was dropped (11.5 mmol) with a pipet. After a stirring for three hours (under nitrogen atmosphere and at room temperature) the reaction residue was roughly purified on a column with dichloromethane/petroleum ether (1:1). To obtain purer substance, the column procedure was re-performed and white solid was collected (0.75 g, 39%). Mp: 97.0-99.0 °C. ^{1}H-NMR (400 MHz, chloroform-d, r. t.) δ (ppm): 3.66 (t, J = 5.7 Hz, 20H, CH_2), 3.87 (s, 10H, CH_2), 4.27 (t, J = 5.7 Hz, 20H, CH_2), 6.98 (s, 10H, PhH), ^{13}C-NMR (100 MHz, chloroform-d, r. t.) δ (ppm): 29.4, 30.7, 68.9, 116.1, 128.4, 149.6, and m/z 1702.5 $[M+H]^+$ (100%). m/z calculated. for $[M+H]^+$ $C_{55}H_{60}Br_{10}O_{10}$, 1702.5938; found 1702.58. Elemental analysis for $C_{55}H_{60}Br_{10}O_{10}$ calcd.: C, 39.32; H, 3.60; found: C, 39.88; H, 3.71. Then, 1,4-bis(2-bromoethoxy)pillar[5]arene and (0.1 mmol) and NaN_3 (1.3 mmol) were dissolved in 10 mL of DMF. After overnight, the raw product was extracted with water/ethyl acetate mixture. The organic phases were collected and dried with Na_2SO_4. FT-IR (N_3: 2071 cm^{-1}). ^{1}H-NMR (400 MHz, chloroform-d, r. t.) δ (ppm): 6.95 (s, 10H, PhH), 4.30 (t, 20H, CH_2), 3.90 (s, 10H, CH_2), 3.72 (t, 20H, CH_2).

The synthesis of P[5]-1

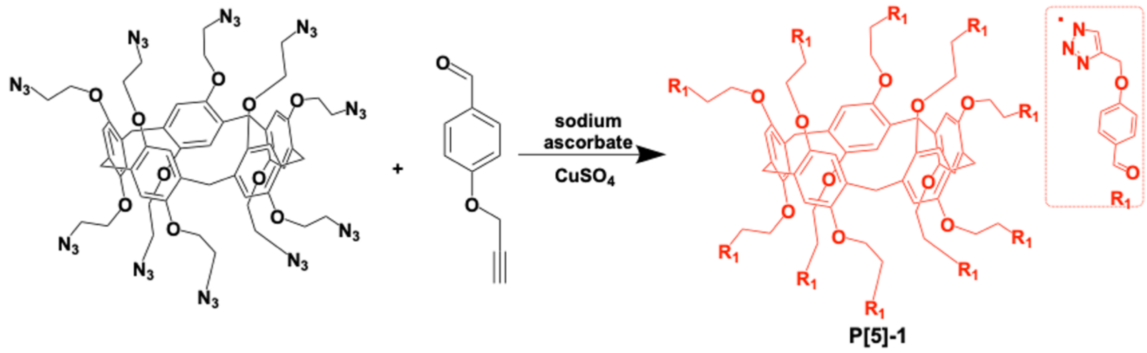

Pillar[5]arene including iodines was prepared such as a literature procedure [1]. Pillar[5]arene including azides (0.1 mmol) and 4-(prop-2-yn-1-yloxy)benzaldehyde (1 mmol), $CuSO_4{\cdot}5H_2O$ (0.01 mmol) and sodium ascorbate (0.02 mmol) in 8 mL of $CH_2Cl_2/H_2O/C_2H_5OH$ solvent mixture (5:5:1) was dissolved and stirred vigorously at room temperature under nitrogen atmosphere. After 72 h, H_2O (15 mL) was added to reaction mixture and the aqueous layer was extracted with CH_2Cl_2 (3×). The organic phases were collected and dried. The raw product was purified with column using solvent mixture (CH_2Cl_2 containing 1.9% of methanol). A white solid material was obtained. ^{1}H NMR (400 MHz, $CDCl_3$): δ=9.95 (s, 10H, HC=O) 7.75-7.70 (m, 30H, PhH), 7.15 (d, 20H, PhH), 7.05 (s, 10 H, PhH), 5.10 (s, 20 H, CH_2), 4.78 (t, 20 H, CH_2), 4.30 (t, 20 H, CH_2), 3.77 (s, 10 H, CH_2). ^{13}C-NMR (100 MHz) δ (ppm): 188.9, 160.8, 137.4, 135.5, 133.2, 131.2, 128.9, 117.3, 116.1, 113.5, 112.4, 74.3, 73.1, 71.4, 52.3, 30.3. Elemental Analysis calculated.: $C_{155}H_{140}N_{30}O_{30}$: C, 64.13; H, 4.86; N, 14.48. found C, 64.45; H, 5.01; N, 14.60.

The synthesis of 1,4-bis(2-iodoethoxy)benzene

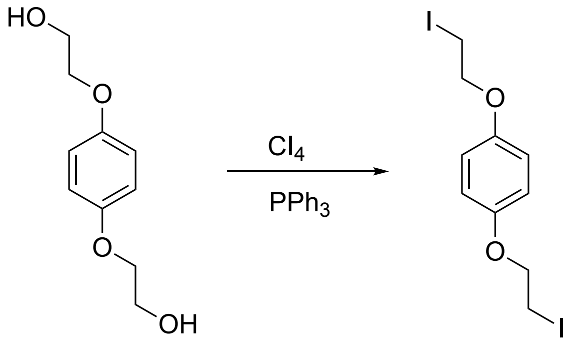

Carbon tetraiodide (20 mmol) was slowly added in small portions to a solution of 1,4-bis(2-hydroxyethoxy)benzene (10 mmol) and triphenylphosphine (10 mmol) in 100 mL of dry acetonitrile at 0 °C. Then, the reaction mixture was stirred at room temperature, and the resulting clear solution was stirred for another 3 h under Ar. 100 g of ice was added to the

reaction mixture, where 1,4-bis(2-iodoethoxy)benzene slowly precipitated as a white solid. The product was collected by vacuum filtration, thoroughly washed with cold methanol/water, 60:40. The white flake-like crystals were dried in desiccator (4.37 g, 87%). M.P.: 92 °C. ^{1}H-NMR (400 MHz, chloroform-d, room temperature) δ (ppm): 6.92 (s, 4H, PhH), 4.33 (t, 4H, CH_2), 3.52 (t, 4H, CH_2). ^{13}C-NMR (100 MHz) δ(ppm): 150.38, 116.10, 75.18, 4.32.

The synthesis of pillar[5]arene including iodines

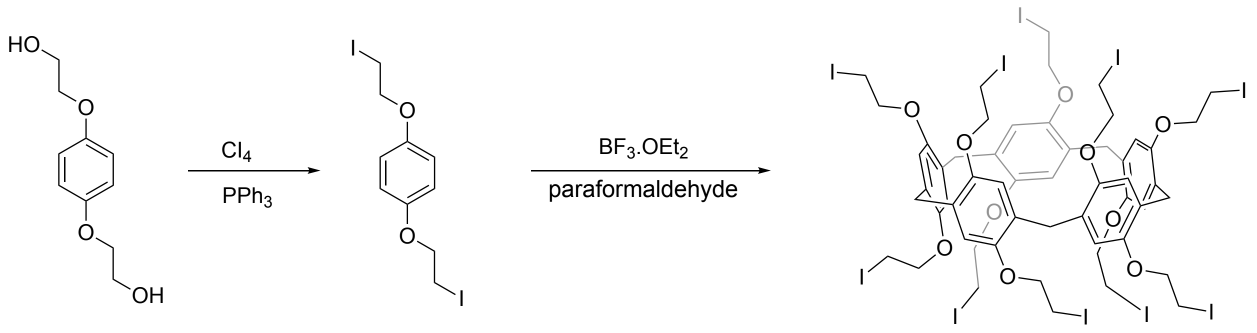

Pillar[5]arene including iodines was prepared according to a literature procedure [1]. $BF_3.OEt_2$ (0.8 g, 6.75 mmol) and paraformaldehyde (18 mmol) in 1,2-dichloroethane (100 mL) was added to a solution of 1,4-bis(2-iodoethoxy)benzene (6.75 mmol) at room temperature. The reaction mixture was stirred for 3 h under N_2 atmosphere. Column chromatography (SiO_2; petroleum ether/CH_2Cl_2, 1:1) was used to purify pillar[5]arene including iodines (0.99 g, 36%). m.p.: 275 °C ^{1}H-NMR (400 MHz, chloroform-d, room temperature) δ (ppm): 6.92 (s, 10H, PhH), 4.33 (t, 20H, CH_2), 3.85 (s, 10H, CH_2), 3.52 (t, 20H, CH_2). ^{13}C-NMR (100 MHz) δ (ppm): 149.88, 125.87, 116.30, 67.38, 29.65, and 3.92. Elemental Analysis calculated.: $C_{55}H_{60}I_{10}O_{10}$: C, 30.72; H, 2.81; found: C, 30.55; H, 3.07.

The synthesis of P[5]-2

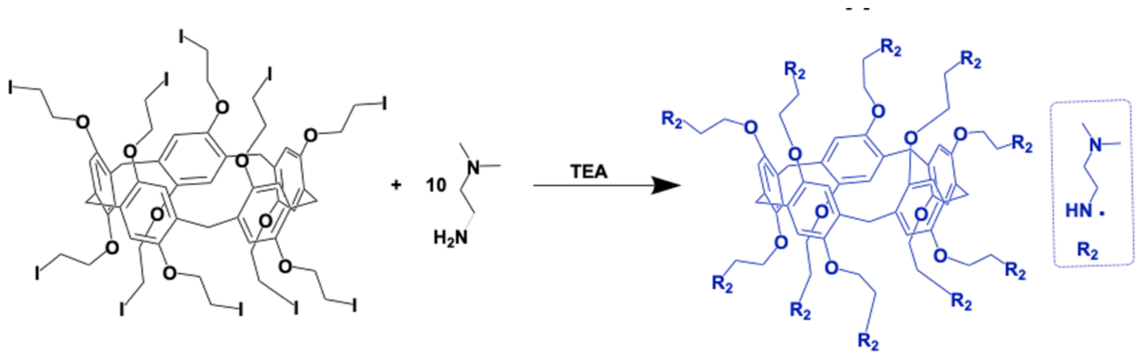

The compounds, 1,4-bis(2-iodoethoxy)benzene and pillar[5]arene including iodines and were synthesized according to following method [2]. To prepare target product, **P[5]-2** was obtained from pillar[5]arene including iodines and N,N-Dimethylethylenediamine with the removal iodine fragments from pillar[5]arene using sodium hydride. For **P[5]-2**: ^{1}H-NMR (400 MHz, chloroform-d, r.t.) δ (ppm): 6.95 (s, 10H, PhH), 4.25 (bs, 10H, NH), 3.75-3.85 (m, 50H, CH_2), 3.33 (t, 20H, CH_2) 2.45 (t, 20H), 2.52 (t, 20H, CH_2), 2.04 (s, 60H, CH_3). ^{13}C-NMR (100 MHz) δ (ppm): 148.4, 128.6, 116.7, 68.8, 62.31, 59.1, 46.8, and 27.2. Elemental Analysis calculated. $C_{95}H_{170}N_{20}O_{10}$: C, 65.11; H, 9.78; N, 15.98. found: C, 65.46; H, 9.99; N, 15.65.

References

[1] Bastug, E.; Kursunlu, A. N.; Guler, E. A fluorescent clever macrocycle: Deca-bodipy bearing a pillar [5]arene and its selective binding of asparagine in half-aqueous medium. Journal of Luminescence 2020, 225, 117343. DOI: https://doi.org/10.1016/j.jlumin.2020.117343.

[2] Kursunlu, A. N.; Acikbas, Y.; Ozmen, M.; Erdogan, M.; Capan, R. Fabrication of LB thin film of pillar[5]arene-2-amino-3-hydroxypyridine for the sensing of vapors. Materials Letters 2020, 267, 127538. DOI: https://doi.org/10.1016/j.matlet.2020.127538.

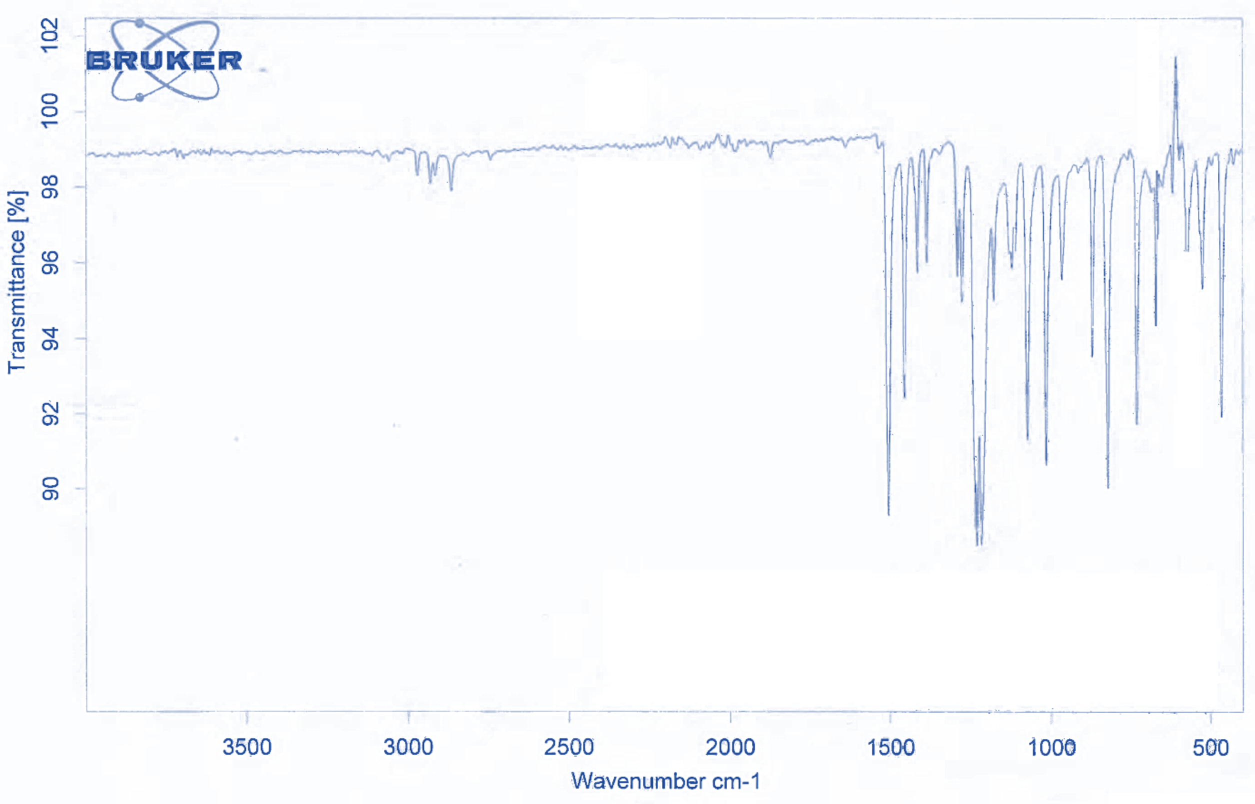

Fig. S1. FT-IR spectrum of pillar[5]arene including iodines.

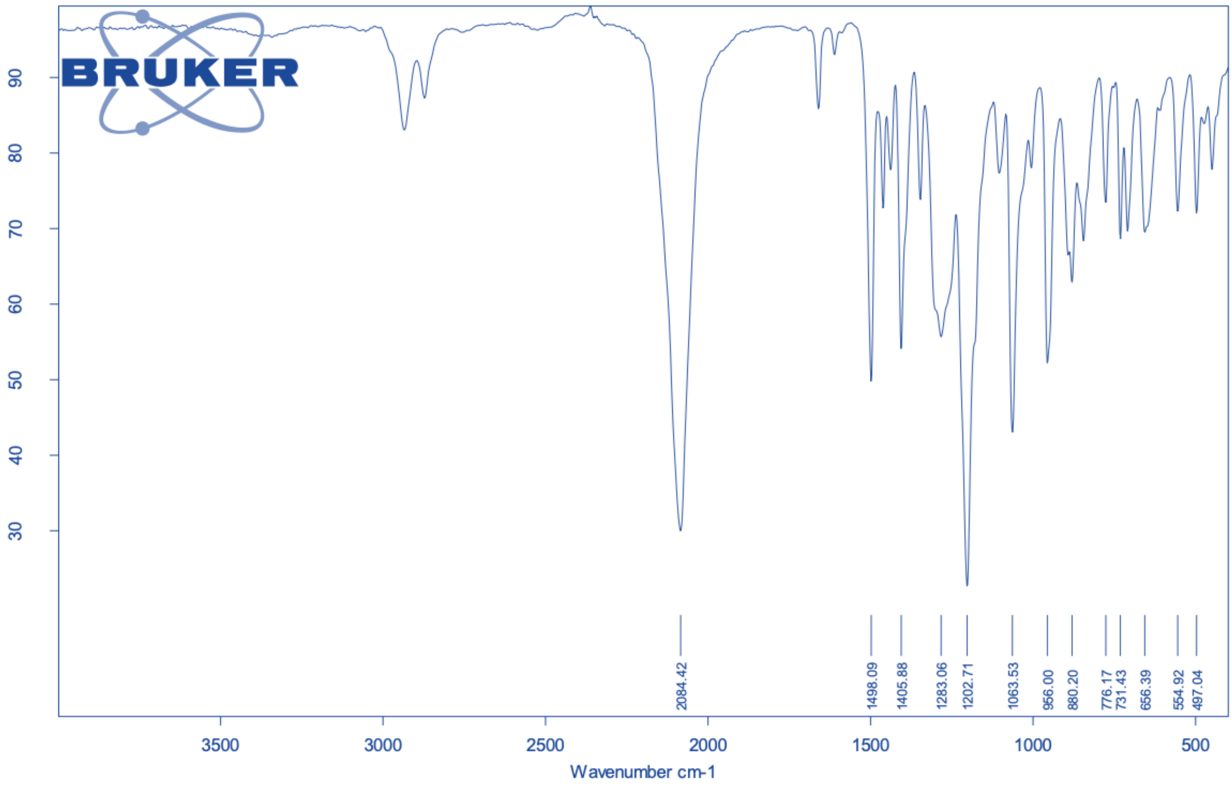

Fig. S2. FT-IR spectrum of pillar[5]arene including azides.

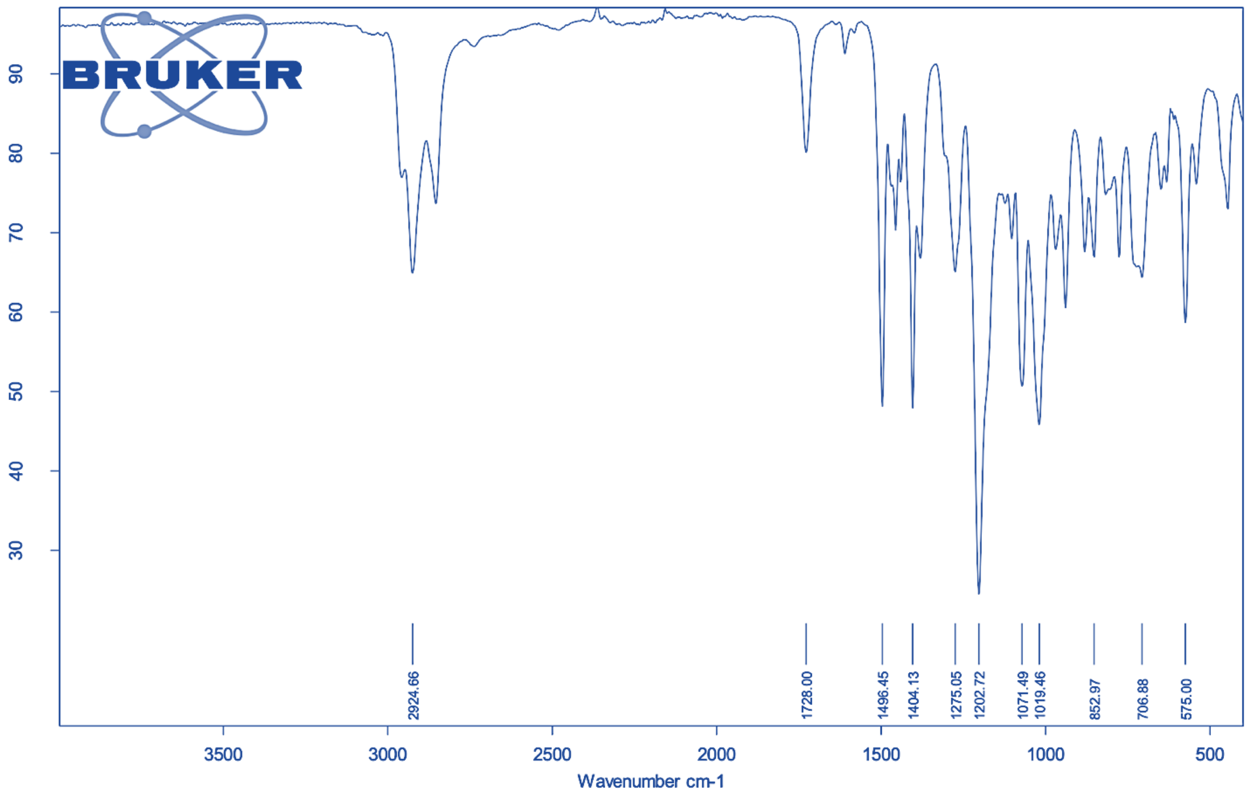

Fig. S3. FT-IR spectrum of **P[5]-1**.

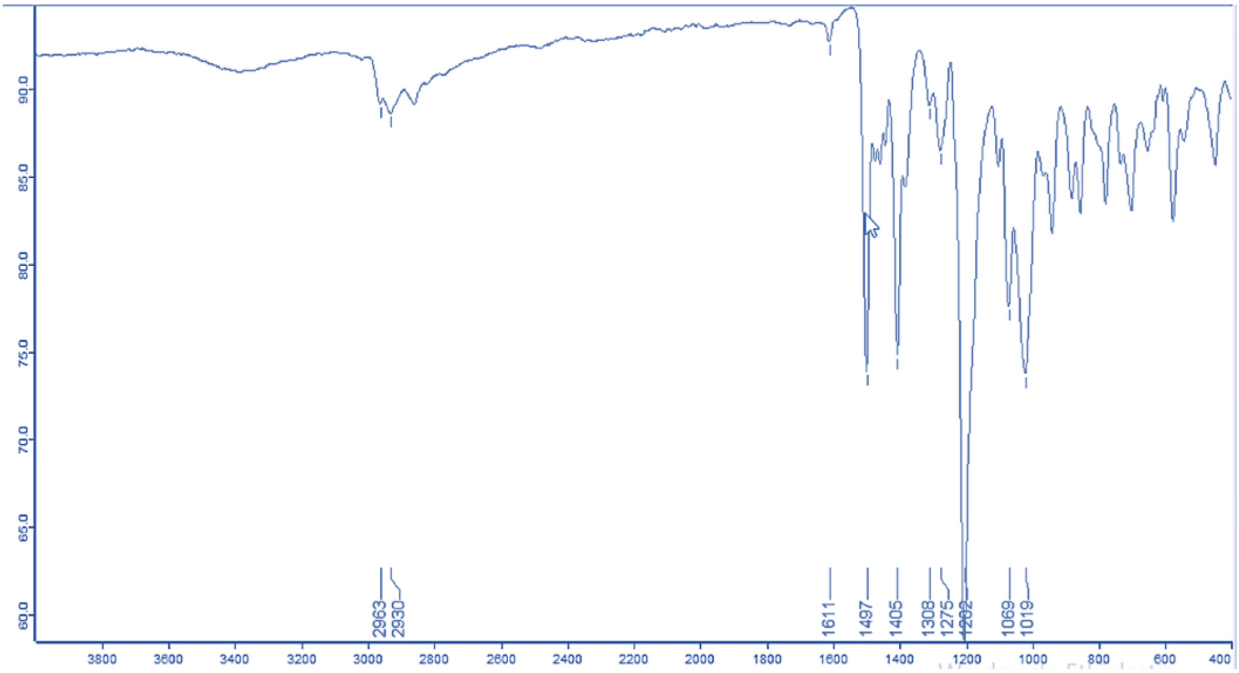

Fig. S4. FT-IR spectrum of **P[5]-2**.

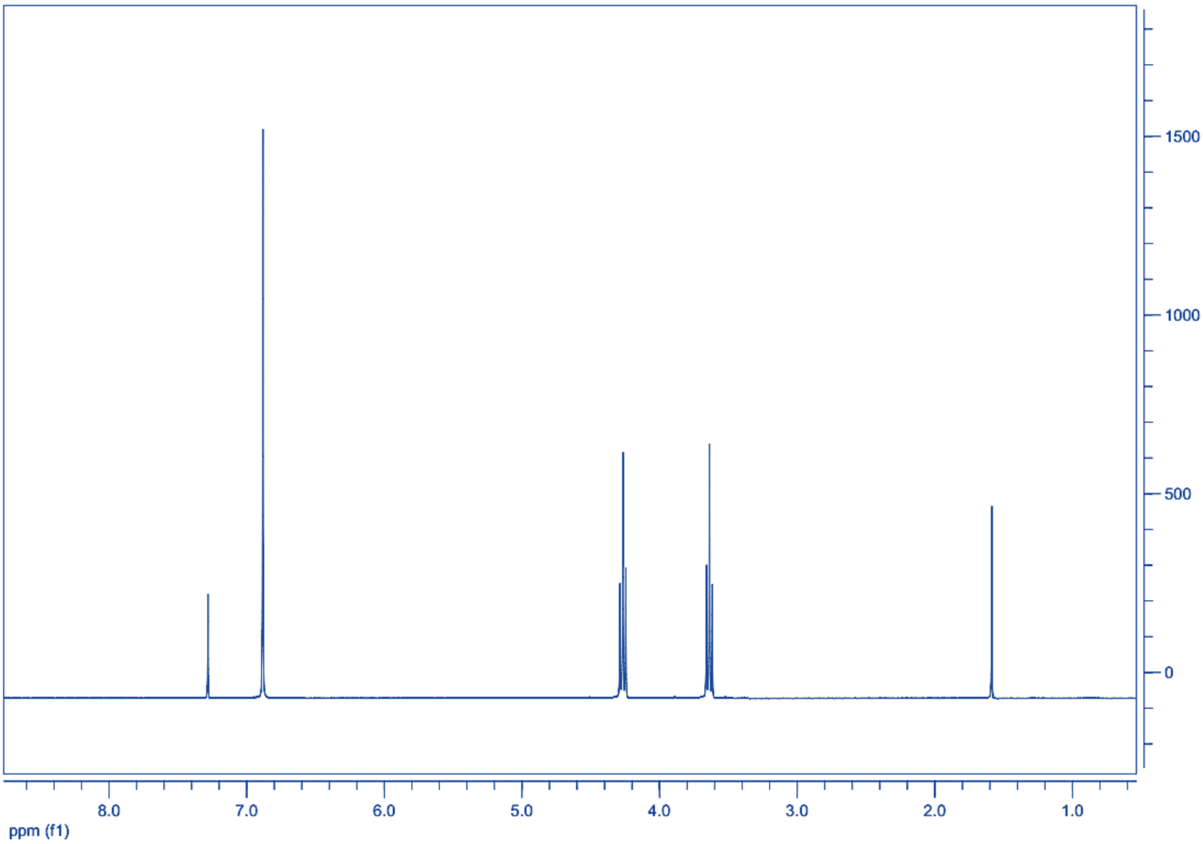

Fig. S5. ^{1}H-NMR spectrum of 1,4-bis(2-iodoethoxy)benzene (25 °C).

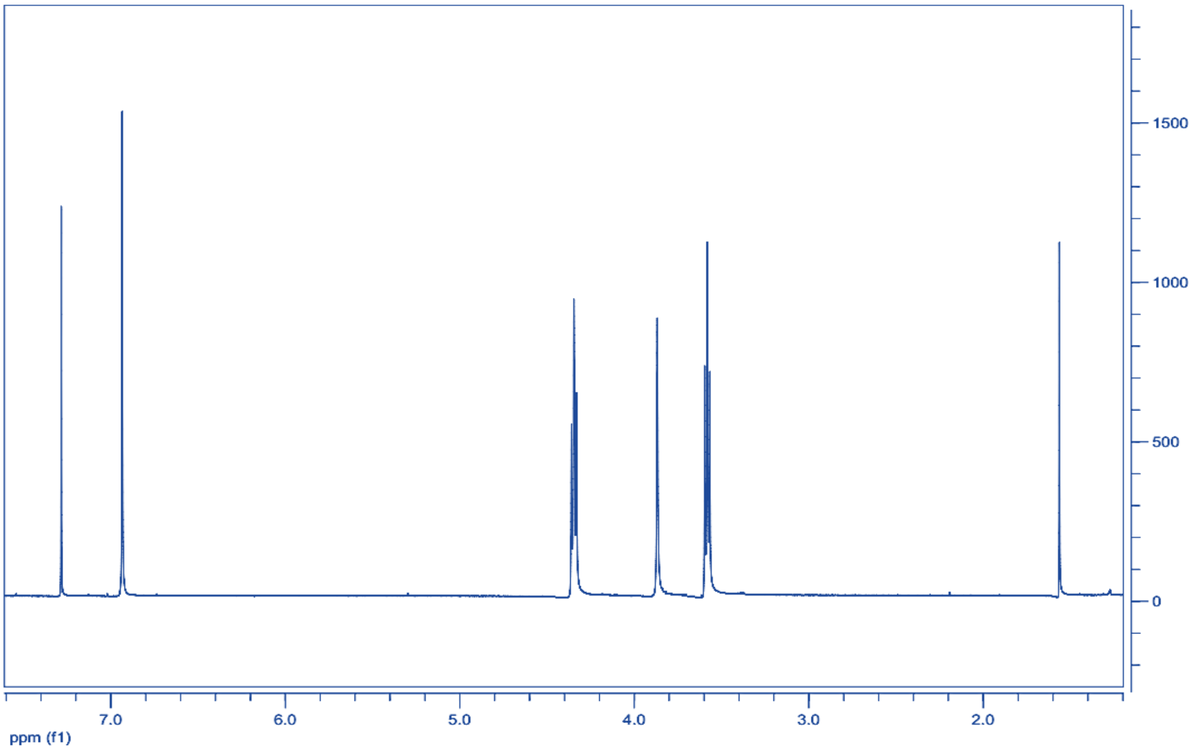

Fig. S6. ^{1}H-NMR spectrum of pillar[5]arene including iodines (25 °C).

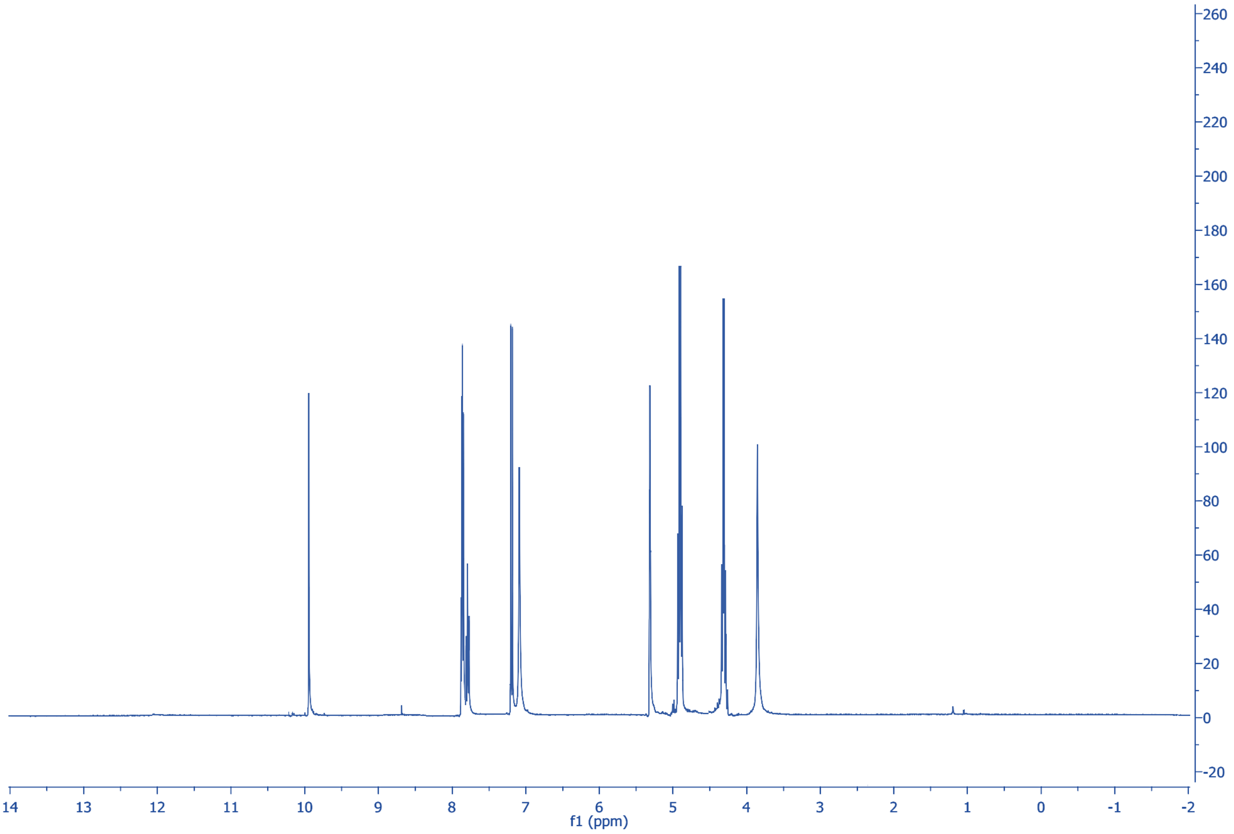

Fig. S7. ^{1}H-NMR spectrum of **P[5]-1**.

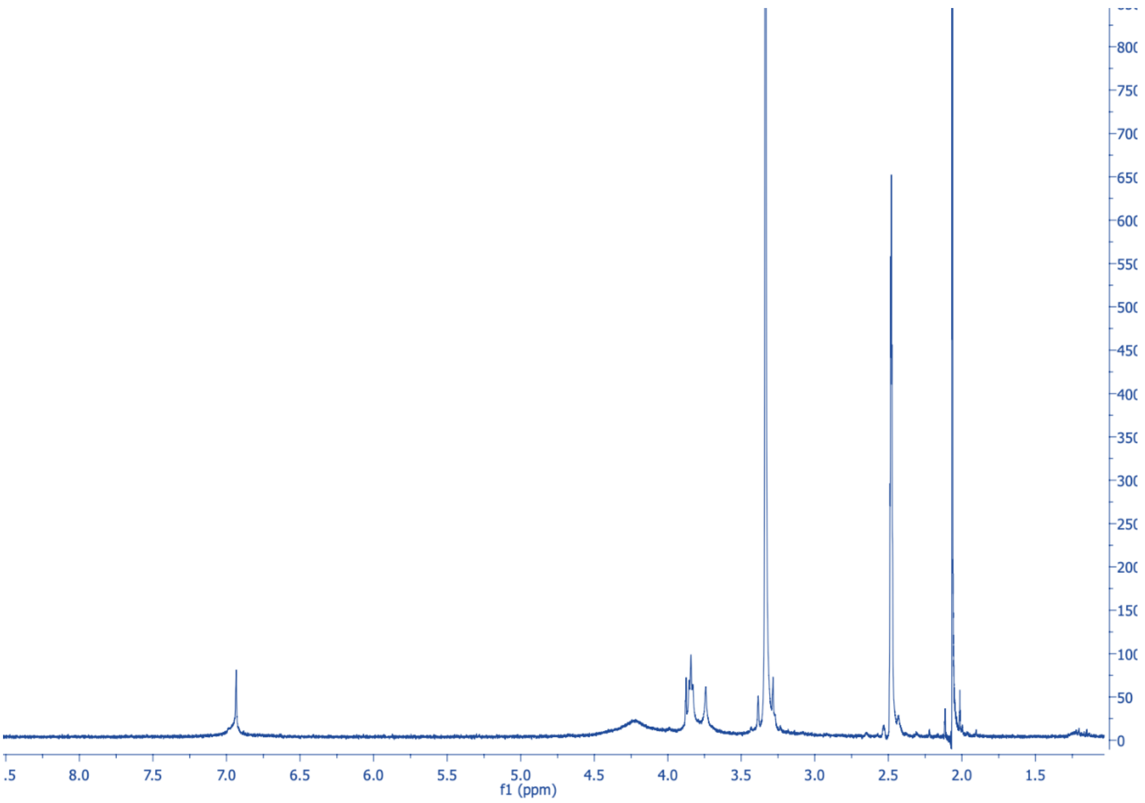

Fig. S8. ^{1}H-NMR spectrum of **P[5]-2**.

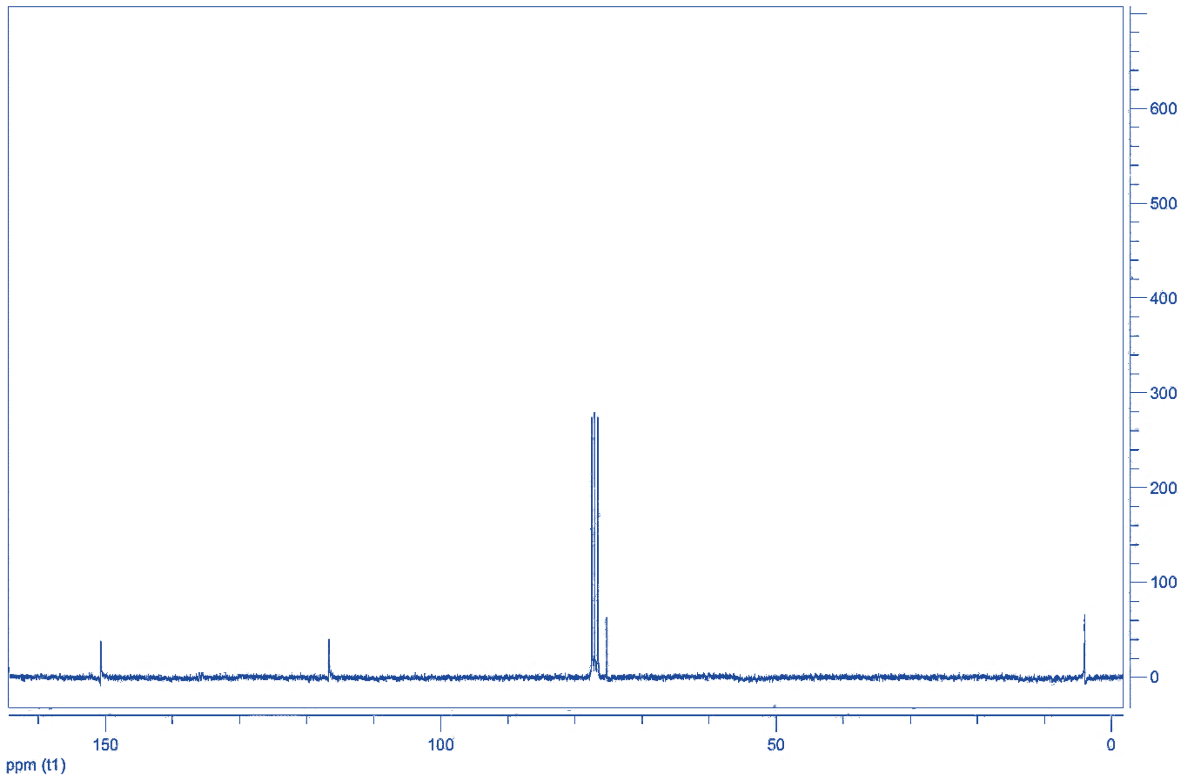

Fig. S9. ^{13}C-NMR spectrum of 1,4-bis(2-iodoethoxy)benzene (25 °C).

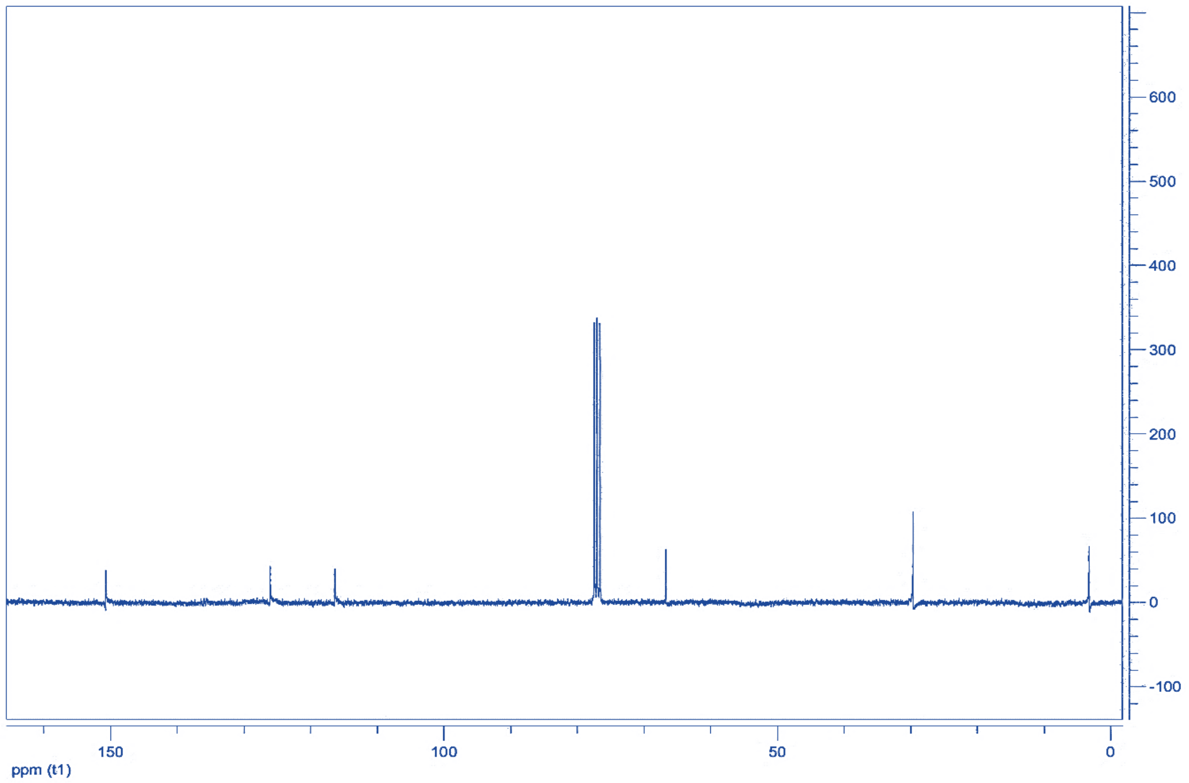

Fig. S10. ^{13}C-NMR spectrum of pillar[5]arene including iodines (25 °C).

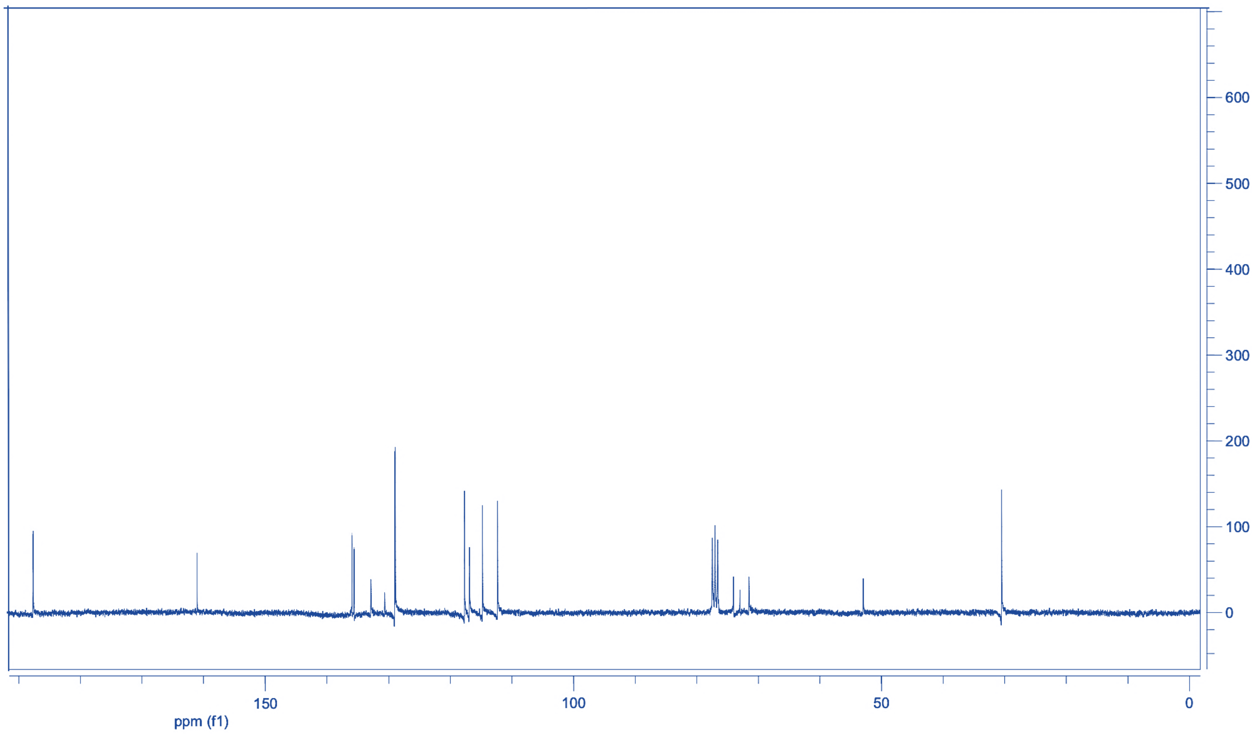

Fig. S11. ^{13}C-NMR spectrum of **P[5]-1** (25 °C).

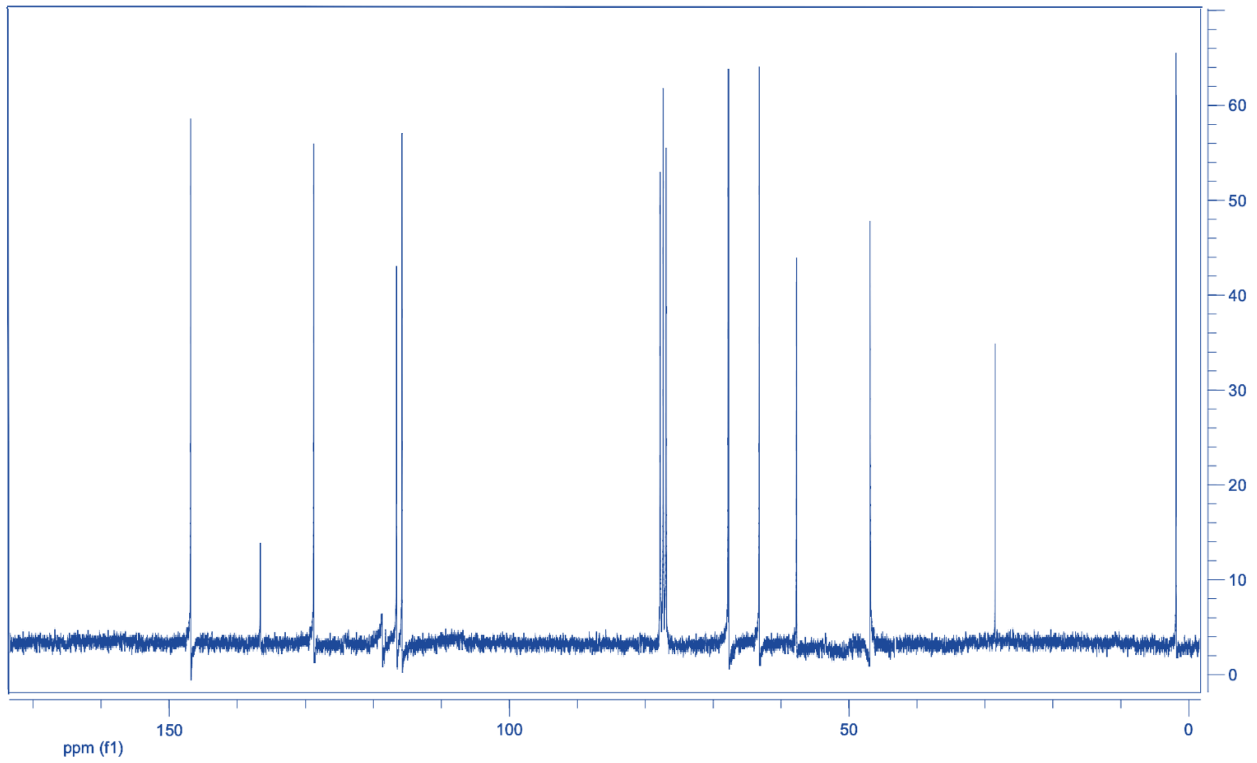

Fig. S12. ^{13}C-NMR spectrum of **P[5]-2** (25 °C).

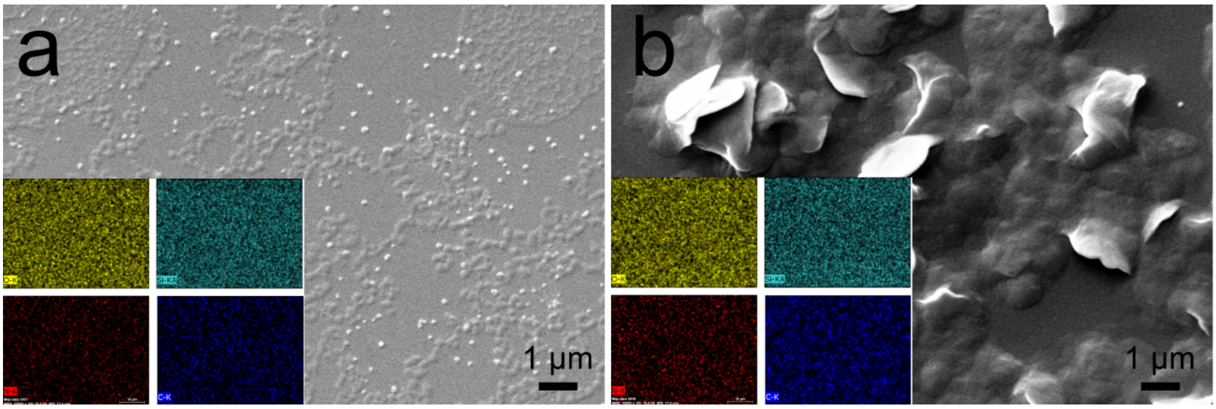

Fig. S13. SEM images of **P[5]-1** coated (a) and **P[5]-2** coated (b) glass substrates (Insets: The elemental mapping images of the following elements: O, Si, N, and C).

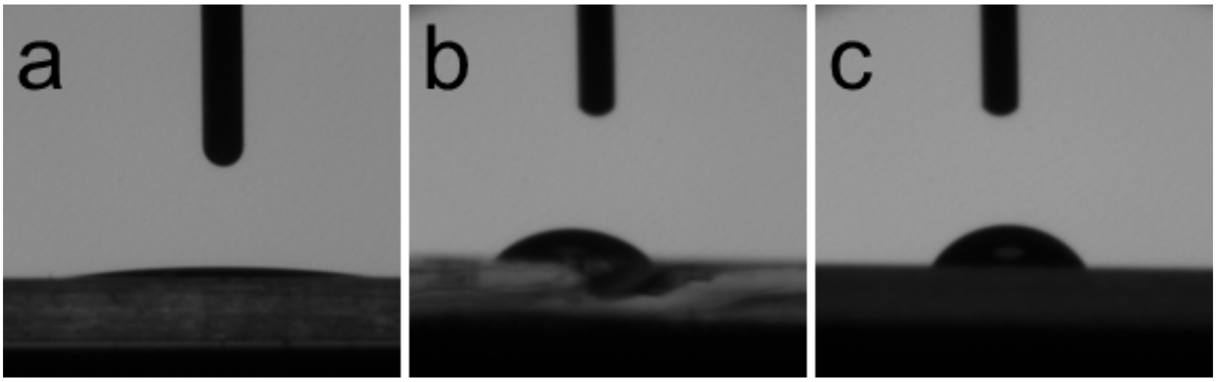

Fig. S14. Contact angle measurements of the bare glass surface (a), **P[5]-1** coated (b) and **P[5]-2** coated (c) glass substrates

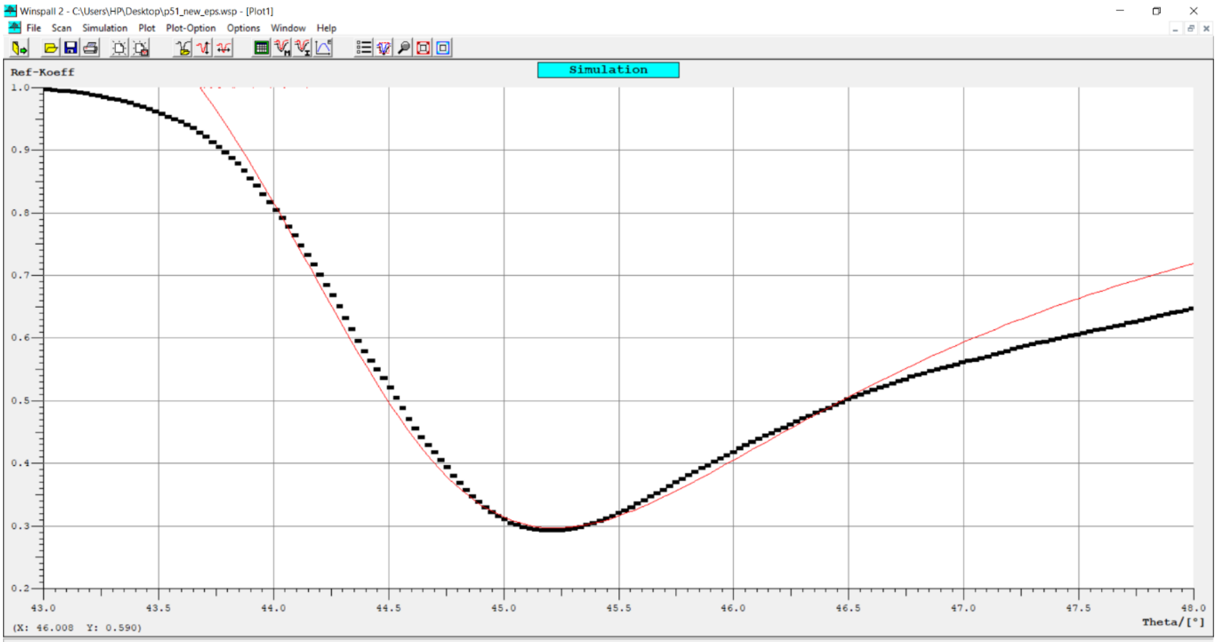

Fig. S15. Winspall fitting graph for **P[5]-1** coated thin film sensor. Experimental data is presented by the dotted line, fitting data is given by red line.

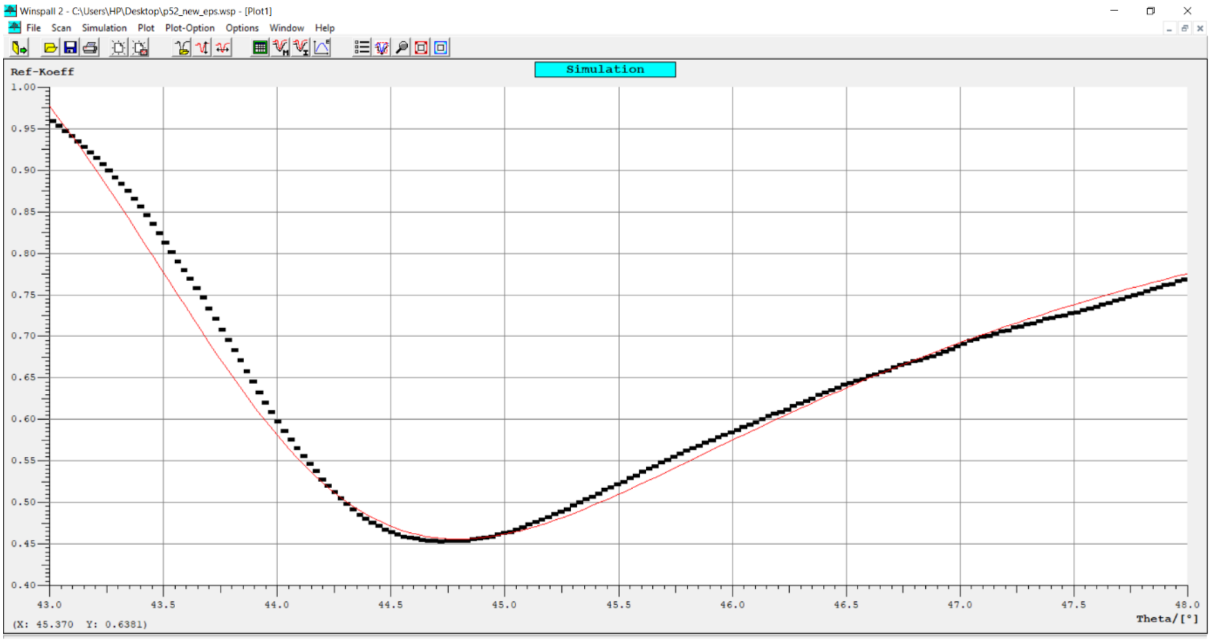

Fig. S16. Winspall fitting graph for **P[5]-2** coated thin film sensor. Experimental data is presented by the dotted line, fitting data is given by red line.

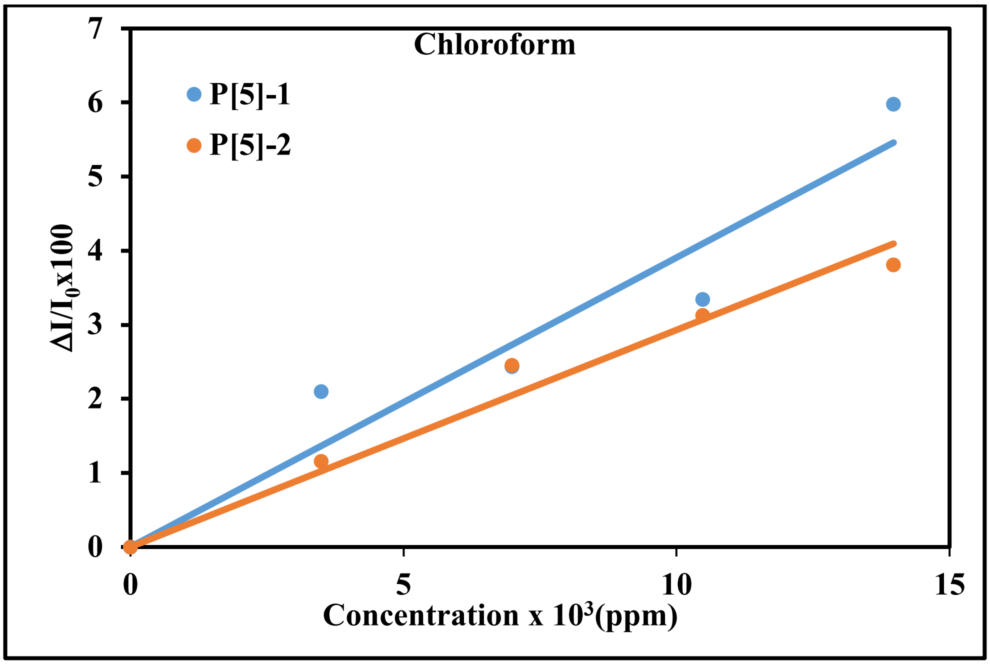

Fig. S17. Calibration curves of the **P[5]-1** and **P[5]-2** thin film sensors to chloroform vapor at increasing concentrations.

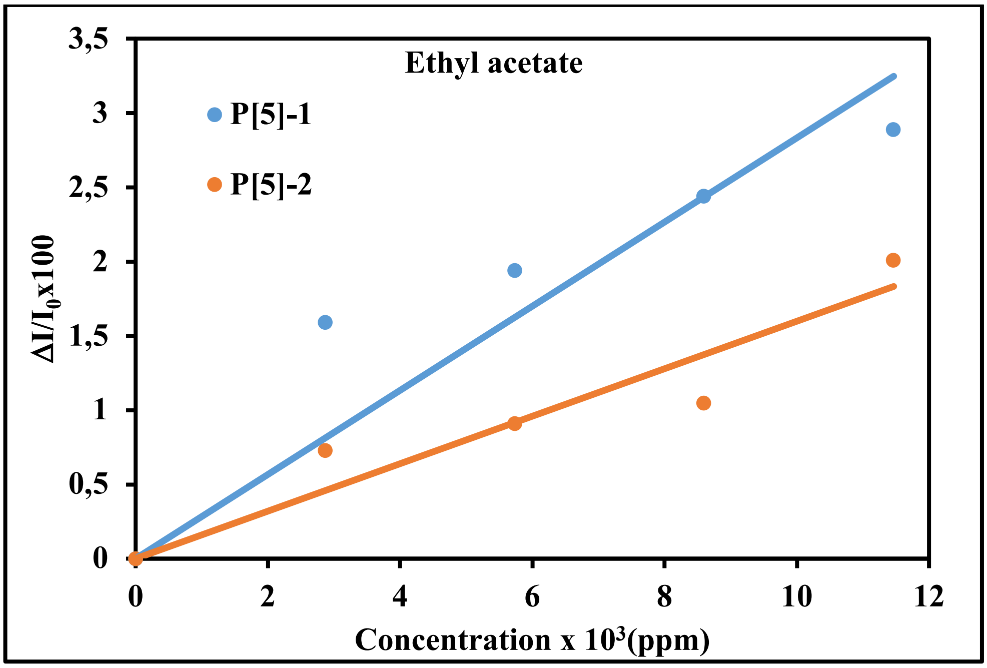

Fig. S18. Calibration curves of the **P[5]-1** and **P[5]-2** thin film sensors to ethyl acetate vapor at increasing concentrations.

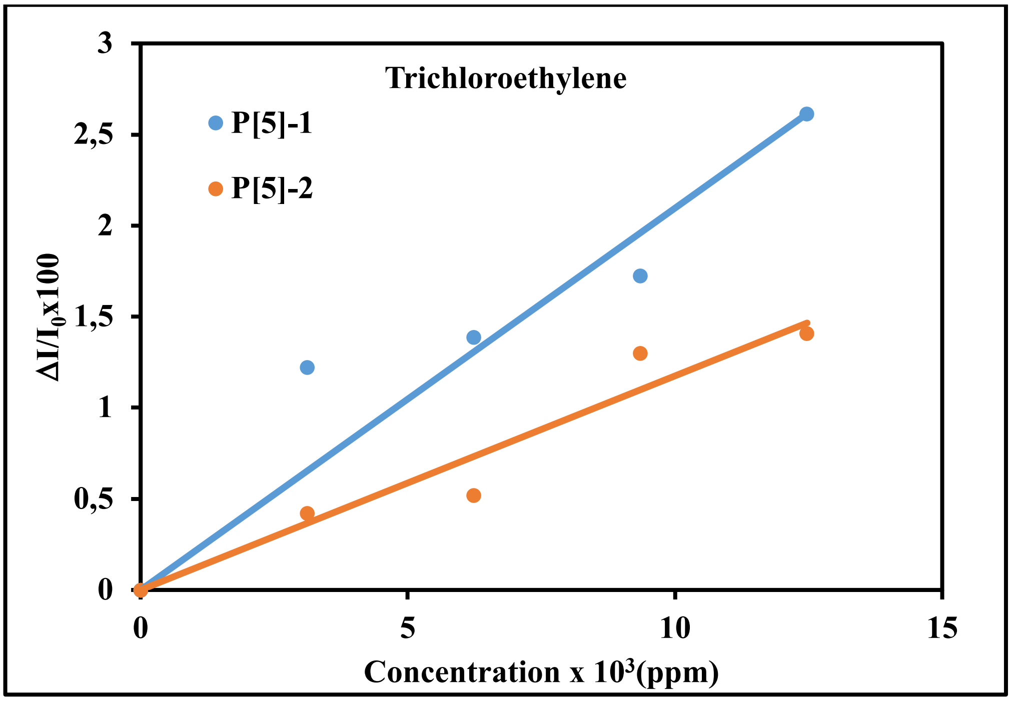

Fig. S19. Calibration curves of the **P[5]-1** and **P[5]-2** thin film sensors to trichloroethylene vapor at increasing concentrations.

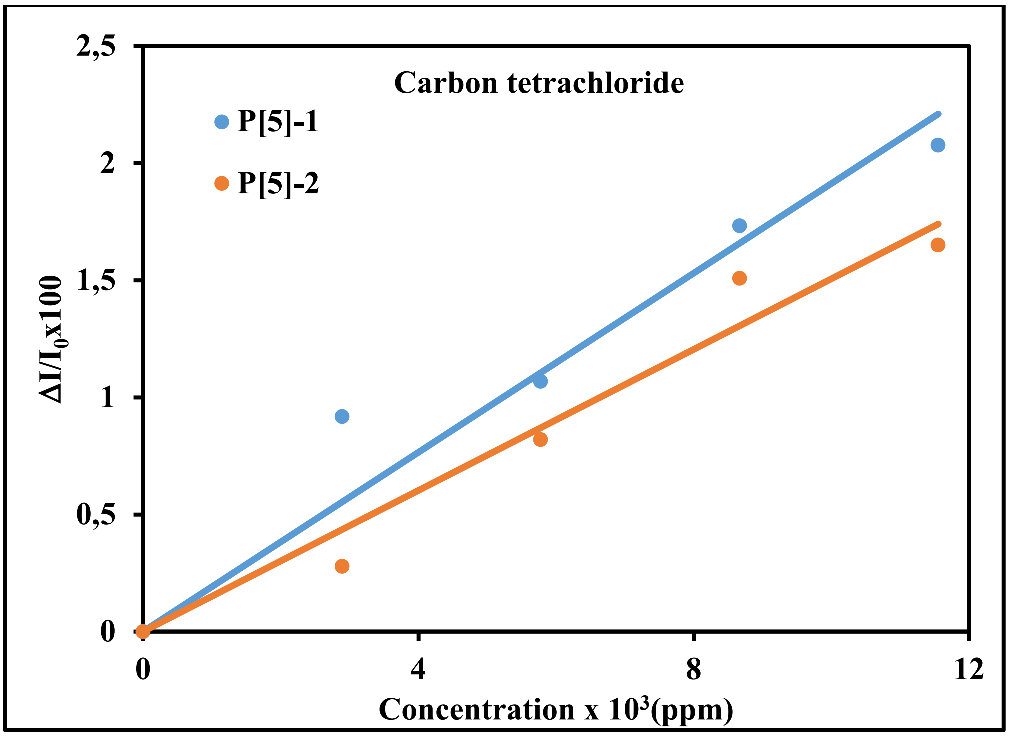

Fig. S20. Calibration curves of the **P[5]-1** and **P[5]-2** thin film sensors to carbon tetrachloride vapor at increasing concentrations.

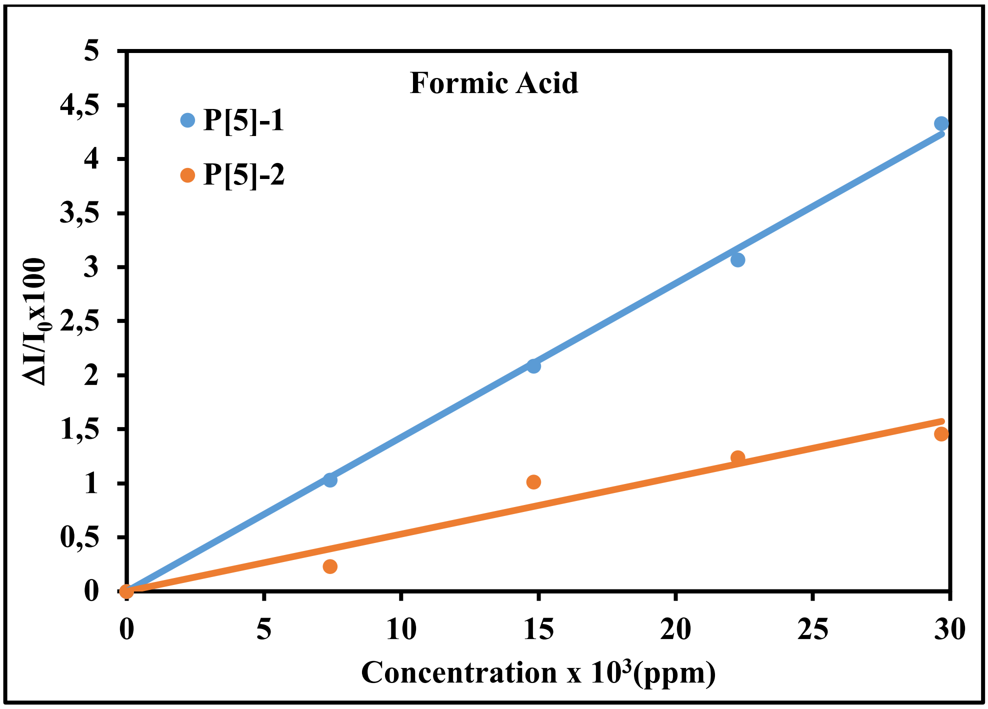

Fig. S21. Calibration curves of the **P[5]-1** and **P[5]-2** thin film sensors to formic acid vapor at increasing concentrations.

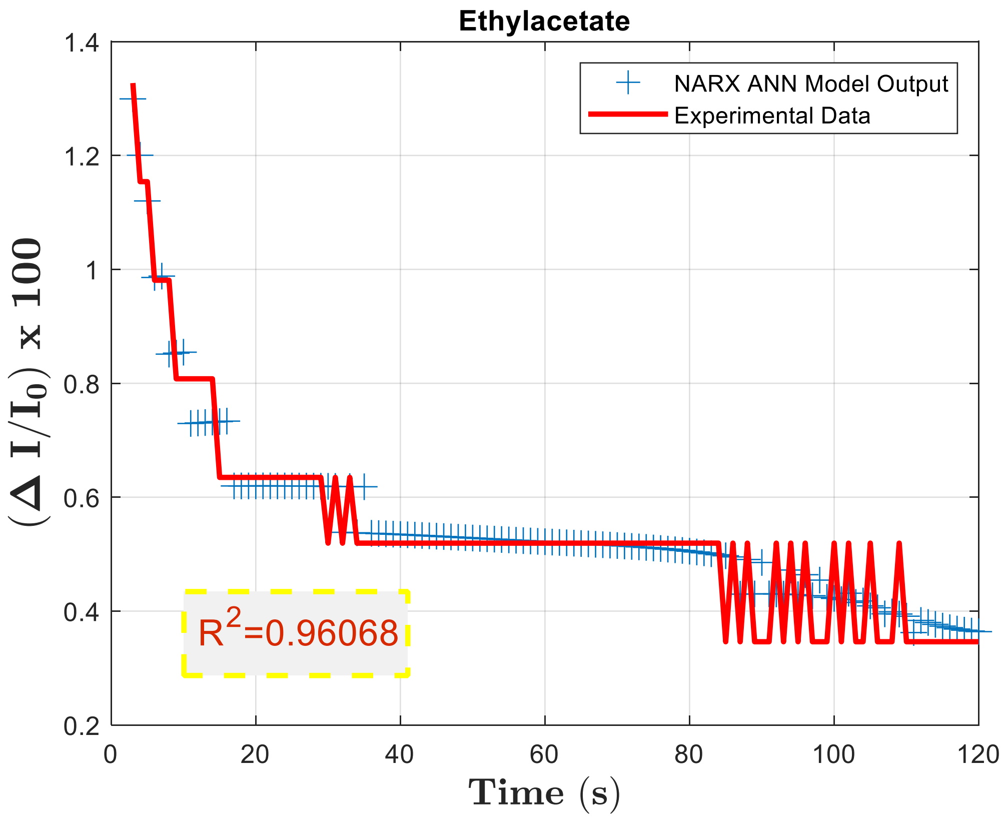

Fig. S22. ANN modelling results of ethyl acetate (**P[5]-1**).

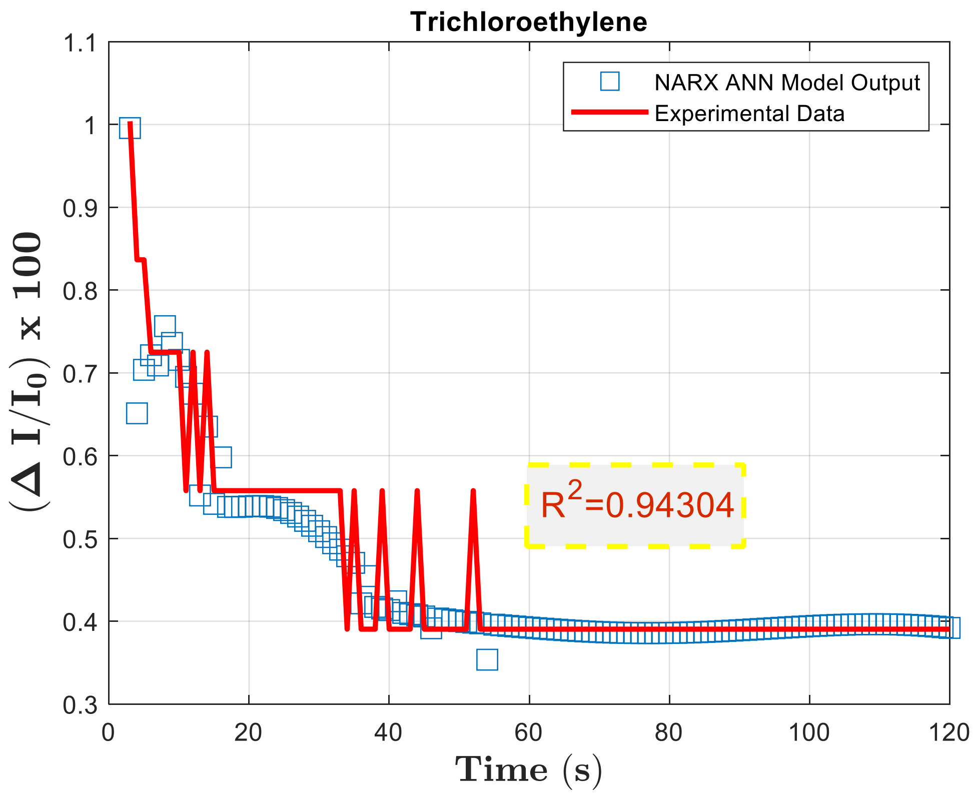

Fig. S23. ANN modelling results of trichloroethylene (**P[5]-1**).

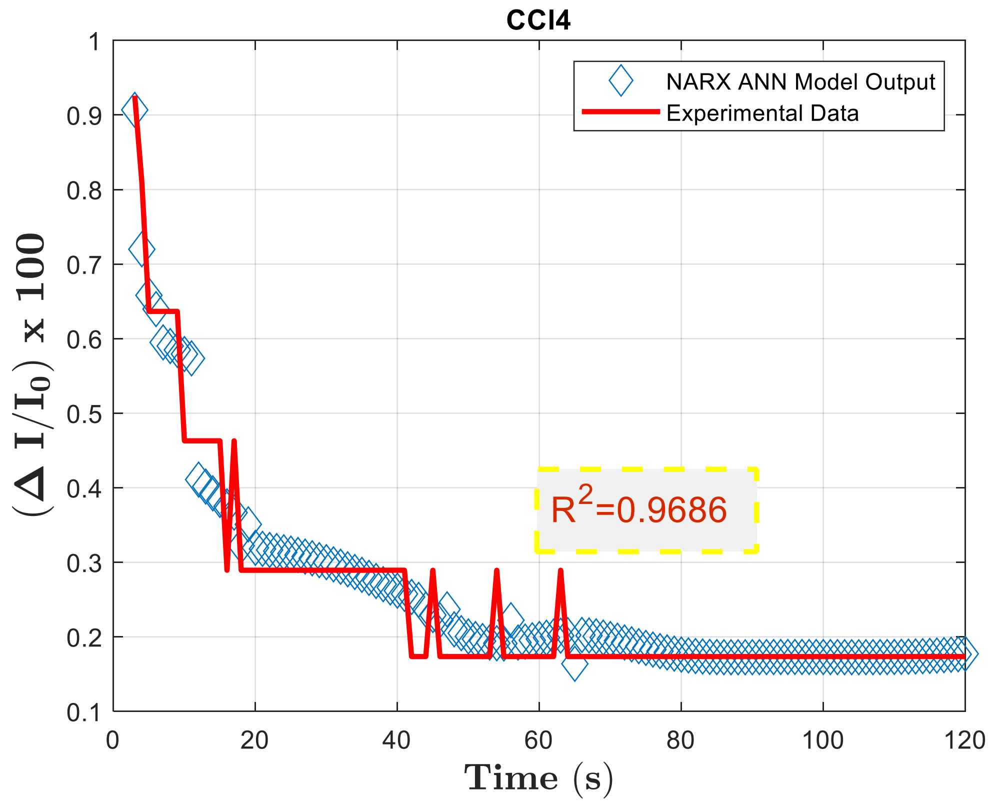

Fig. S24. ANN modelling results of carbon tetrachloride (**P[5]-1**).

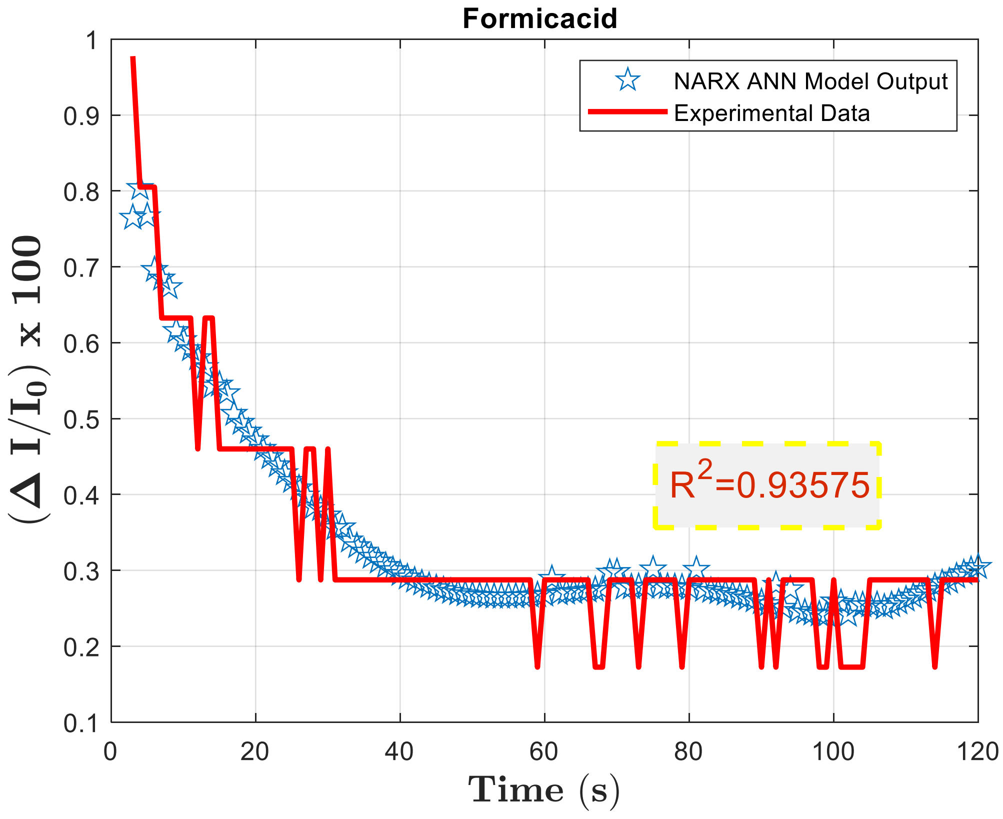

Fig. S25. ANN modelling results of carbon formic acid (**P[5]-1**).

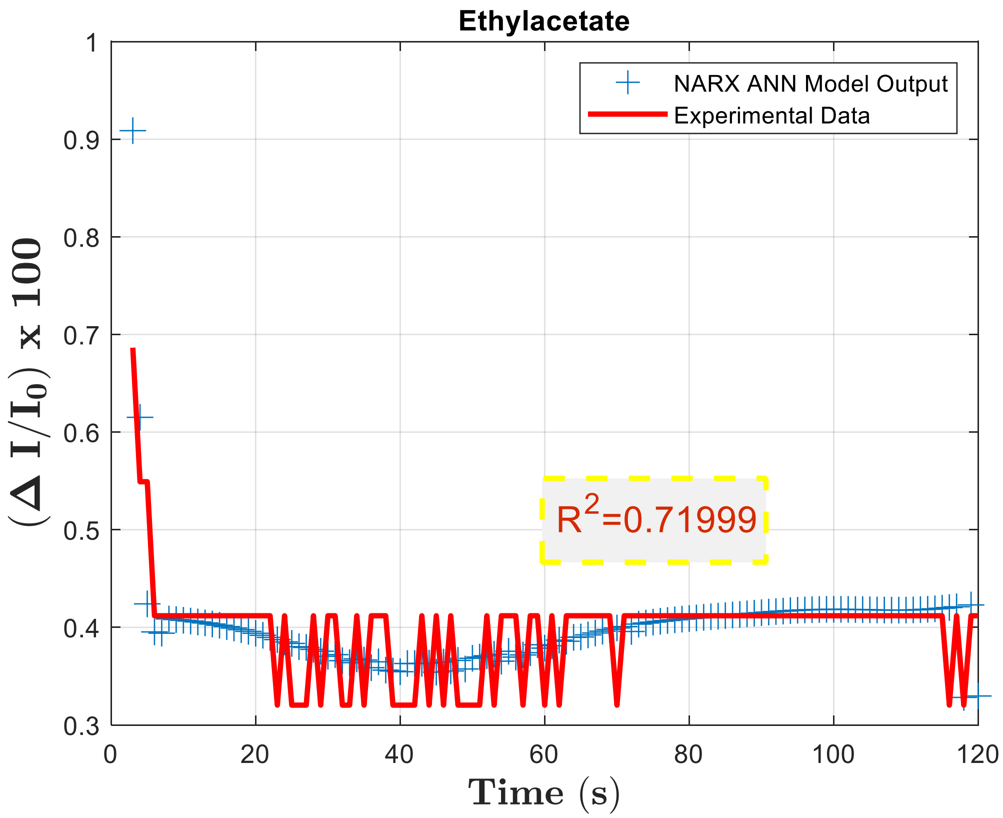

Fig. S26. ANN modelling results of ethyl acetate (**P[5]-2**).

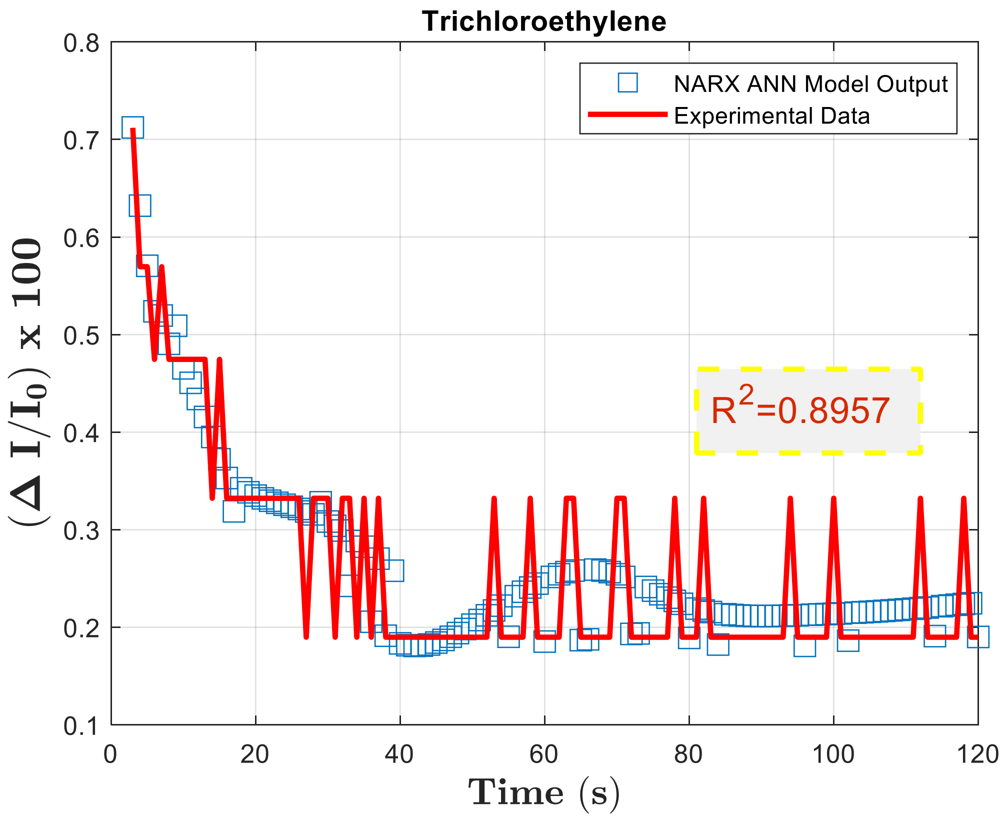

Fig. S27. ANN modeling results of trichloroethylene (**P[5]-2**).

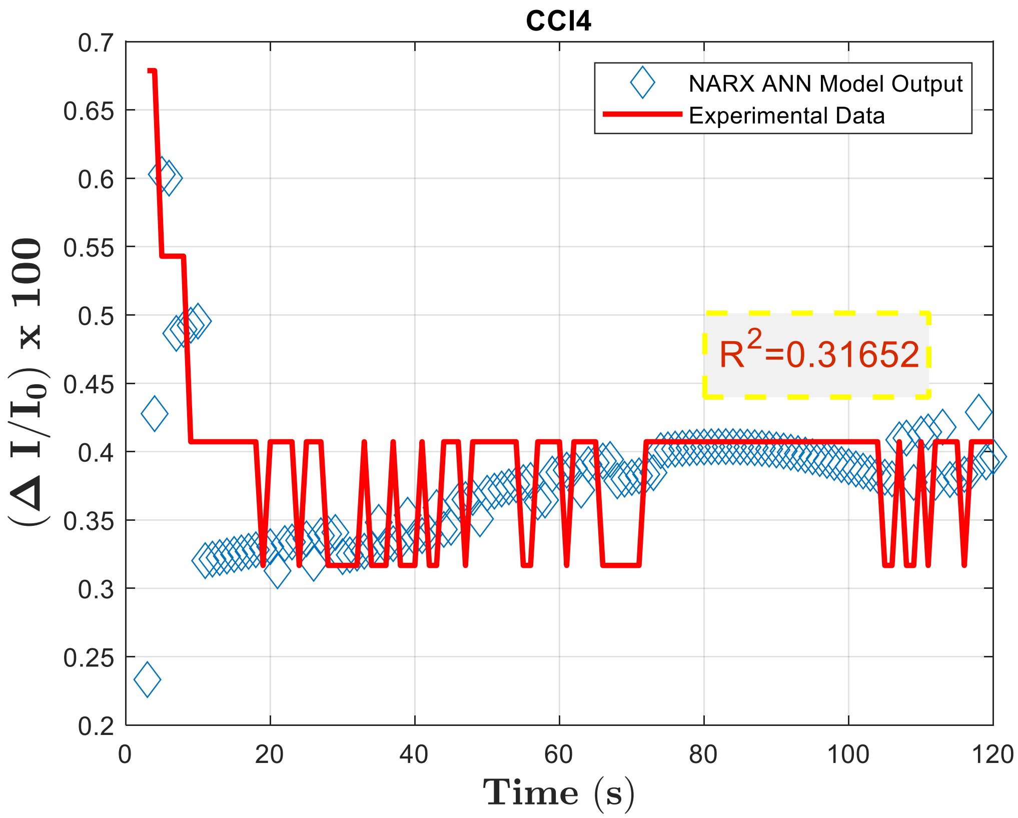

Fig. S28. ANN modelling results of carbon tetrachloride (**P[5]-2**).

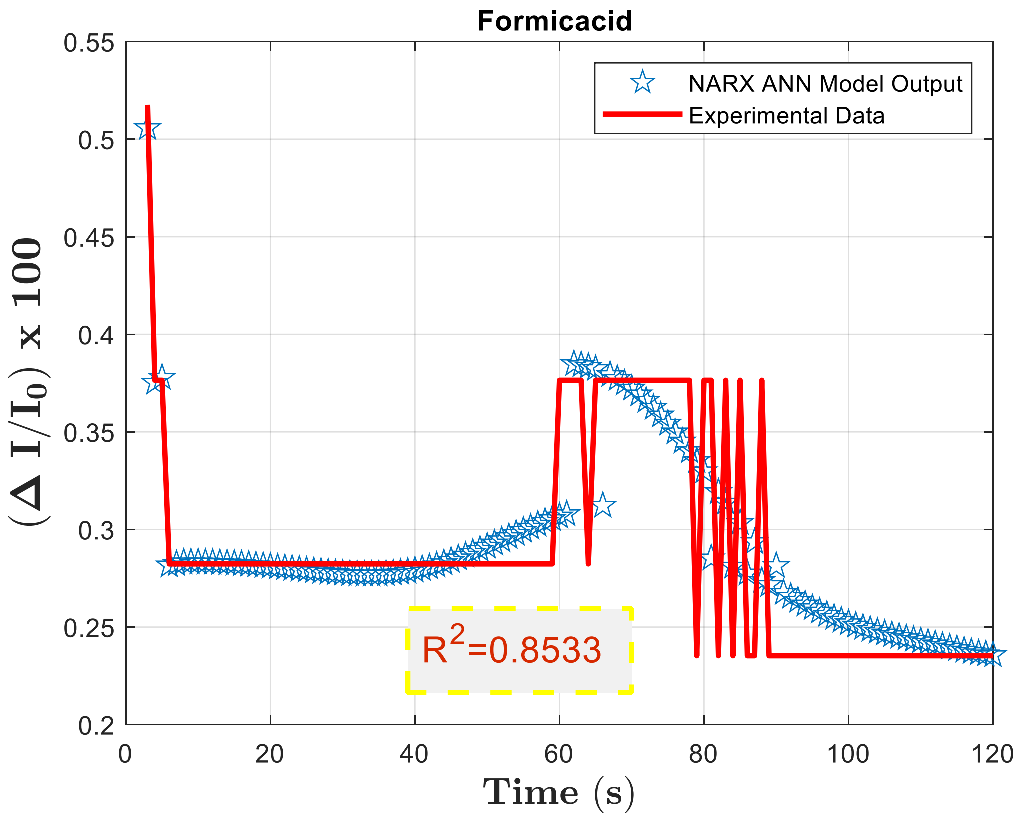

Fig. S29. ANN modelling results of carbon formic acid (**P[5]-2**).
